# Neopentyl glycol-based radiohalogen-labeled amino acid derivatives for cancer radiotheranostics

**DOI:** 10.1186/s41181-024-00244-4

**Published:** 2024-02-26

**Authors:** Yuta Kaizuka, Hiroyuki Suzuki, Tadashi Watabe, Kazuhiro Ooe, Atsushi Toyoshima, Kazuhiro Takahashi, Koichi Sawada, Takashi Iimori, Yoshitada Masuda, Takashi Uno, Kento Kannaka, Tomoya Uehara

**Affiliations:** 1 Graduate School of Pharmaceutical Sciences, Chiba University, 1-8-1 Inohana, Chuo-Ku, Chiba, 260-8675 Japan; 2 Department of Nuclear Medicine and Tracer Kinetics, Graduate School of Medicine, Osaka University, 2-2 Yamadaoka, Suita, Osaka 565-0871 Japan; 3 Institute for Radiation Sciences, Osaka University, 1-1 Machikaneyama, Toyonaka, Osaka 560-0043 Japan; 4 Advanced Clinical Research Center, Fukushima Medical University, 1 Hikarigaoka, Fukushima, 960-1295 Japan; 5 Department of Radiology, Chiba University Hospital, 1-8-1 Inohana, Chuo-Ku, Chiba, 260-8677 Japan; 6 Diagnostic Radiology and Radiation Oncology, Graduate School of Medicine, Chiba University, 1-8-1 Inohana, Chuo-Ku, Chiba, 260-8670 Japan

**Keywords:** Astatine-211, Theranostics, Neopentyl, Radiohalogen, Amino acid

## Abstract

**Background:**

L-type amino acid transporter 1 (LAT1) is overexpressed in various cancers; therefore, radiohalogen-labeled amino acid derivatives targeting LAT1 have emerged as promising candidates for cancer radiotheranostics. However, ^211^At-labeled amino acid derivatives exhibit instability against deastatination in vivo, making it challenging to use ^211^At for radiotherapy. In this study, radiohalogen-labeled amino acid derivatives with high dehalogenation stability were developed.

**Results:**

We designed and synthesized new radiohalogen-labeled amino acid derivatives ([^211^At]At-NpGT, [^125^I]I-NpGT, and [^18^F]F-NpGT) in which L-tyrosine was introduced into the neopentyl glycol (NpG) structure. The radiolabeled amino acid derivatives were recognized as substrates of LAT1 in the in vitro studies using C6 glioma cells. In a biodistribution study using C6 glioma-bearing mice, these agents exhibited high stability against in vivo dehalogenation and similar biodistributions. The similarity of [^211^At]At-NpGT and [^18^F]F-NpGT indicated that these pairs of radiolabeled compounds would be helpful in radiotheranostics. Moreover, [^211^At]At-NpGT exhibited a dose-dependent inhibitory effect on the growth of C6 glioma-bearing mice.

**Conclusions:**

[^211^At]At-NpGT exhibited a dose-dependent inhibitory effect on the tumor growth of glioma-bearing mice, and its biodistribution was similar to that of other radiohalogen-labeled amino acid derivatives. These findings suggest that radiotheranostics using [^18^F]F-NpGT and [^123/131^I]I-NpGT for diagnostic applications and [^211^At]At-NpGT and [^131^I]I-NpGT for therapeutic applications are promising.

**Supplementary Information:**

The online version contains supplementary material available at 10.1186/s41181-024-00244-4.

## Background

Because of their abnormal proliferation, many malignant tumor cells exhibit higher amino acid and glucose uptake than normal cells. Consequently, several amino acids and glucose transporters are more highly expressed in cancer cells than in normal cells. Among these, L-type amino acid transporter 1 (LAT1), an isoform of the L-system, a Na^+^-independent neutral amino acid transporter, is highly expressed in various types of human cancers and plays a vital role in cancer growth and survival(Hafliger and Charles 2019; Kandasamy et al. 2018). Consequently, radionuclide-labeled tracers targeting LAT1 may be helpful in imaging and radiotherapy of a wide range of cancers.

**Fig. 1:**
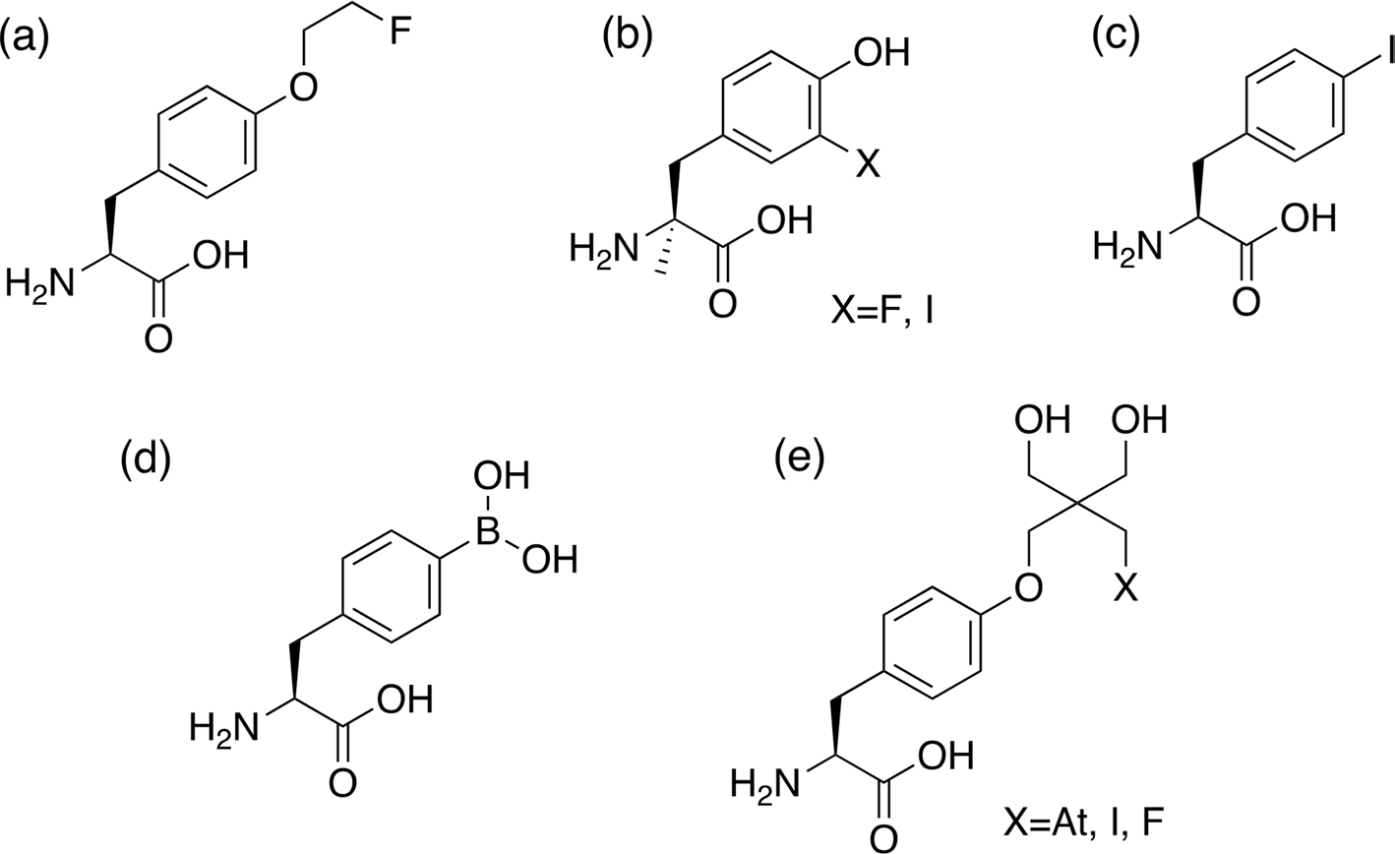
Chemical structure of L-tyrosine and L-phenylalanine derivatives. **a** fluoroethyl-L-tyrosine (FET), **b** 3-fluoro-α-methyl L-tyrosine or 3-iodo-α-methyl L-tyrosine (FMT or IMT), **c** 4-iodo-L-phenylalanine (IPA), **d** 4-borono-L-phenylalaine (BPA), and **e** neopentyl glycol derivatives evaluated in this study (At-NpGT, I-NpGT, F-NpGT)

Many radiohalogen-containing amino acid derivatives have been developed for single-photon emission computed tomography (SPECT) and positron emission tomography (PET) (Fig. 1) (Kratochwil et al. 2014; Morita et al. 2013; Hellwig et al. 2008; Kersemans et al. 2006). In addition, radiotherapy using β^−^-emitting radionuclide ^131^I, *para*-[^131^I]-iodo-L-phenylalanine ([^131^I]-IPA) was also being investigated as a therapeutic agent and had shown efficacy in cancer treatment in clinical trials (Baum et al. 2011). Recent reports have often suggested that radiolabeled compounds containing α-ray emitting radionuclides showed better therapeutic effects than those using β^−^-emitting radionuclides (McDevitt et al. 2018; Morgenstern et al. 2020; Sgouros et al. 2020). Therefore, amino acid derivatives containing astatine-211 (^211^At), a radionuclide that emits α-ray, have been studied (Watabe et al. 2020; Ohshima et al. 2020; Kaneda-Nakashima et al. 2021). These ^211^At-labeled compounds have shown therapeutic effects in mouse models. However, ^211^At-labeled amino acid derivatives are unstable in vivo and undergo deastatination, reportedly owing to weak carbon-astatine bond strength. The release of ^211^At from these compounds and its subsequent loss from tumor cells leads to decreased therapeutic efficacy. This phenomenon is unsuitable for radiotheranostics because the biodistribution of radioactivity after the injection of ^211^At-labeled amino acid derivatives is different from that of diagnostic radiolabeled amino acid derivatives that are stable in vivo. In other words, ^211^At-labeled amino acid derivatives that are stable against deastatination in vivo would be useful radiopharmaceuticals for radiotherapy.

We recently reported that the neopentyl glycol (NpG) structure is an effective scaffold for labeling radiohalogens, including ^211^At (Suzuki et al. 2021). Generally, alkyl halides (especially heavy halogens) are unstable against dehalogenation in vivo; however, radiohalogen-labeled compounds using the NpG structure show high stability against dehalogenation in vivo owing to high steric hindrance and resistance to P450 metabolism. Therefore, we planned to synthesize radiohalogen-labeled amino acid derivatives using NpG as the radiohalogen-labeling moiety. According to the structures of radiolabeled amino acid derivatives such as [^18^F]-fluoroethyl-L-tyrosine ([^18^F]-FET), modification of the hydroxyl group of phenol in L-tyrosine is expected to be acceptable for substrate recognition by LAT1. Based on these considerations, we designed a radiohalogen-labeled amino acid derivative by introducing an NpG structure into the hydroxyl group of the phenol in L-tyrosine. Because the NpG group is a valuable labeling moiety for all radiohalogen elements, radiohalogen-labeled amino acid derivatives ([^211^At]At-NpGT, [^125^I]I-NpGT, and [^18^F]F-NpGT; Fig. 1) were synthesized and characterized for their LAT1-specific cellular uptake and biodistribution in tumor-bearing mice. Furthermore, their usefulness as radiotheranostic pharmaceuticals was evaluated.

## Methods

The preparation of [^211^At]At-NpGT, [^125^I]I-NpGT, and [^18^F]F-NpGT and the analytical methods using RP-TLC and RP-HPLC are described in the Additional file 1.

### Cellular uptake study

The rat glial cell line, C6 (RCB2854), was provided by the RIKEN BRC through the National Bio-Resource Project of the MEXT/AMED, Japan. The C6 glioma cells were trypsinized and suspended in 10% Fetal Bovine Serum (FBS) (Cosmo Bio Co., LTD., Tokyo, Japan) /RPMI1640 (Nacalai Tesque, Kyoto, Japan) medium at a density of 5 × 10^5^ cells/tube. Each tube was centrifuged at 300 × *g* for 5 min. The supernatant was discarded, and the cells were washed with 10 mM HEPES/Hanks’ balanced salt solution (HBSS) (Nacalai Tesque) (1 mL × 2). The cells were resuspended in 10 mM HEPES/HBSS (500 µL). After preincubation at 37 ˚C for 5 min, 20 μL of 10 mM HEPES/HBSS containing either [^125^I]I-NpGT (3.7 kBq), [^211^At]At-NpGT (30 kBq), or [^18^F]F-NpGT (40 kBq) was added and incubated at 37 ˚C for 1, 10, and 30 min. The uptake of radiolabeled amino acid derivatives was terminated by adding 1000 μL of ice-cold PBS (Nacalai Tesque), and the mixture was allowed to stand for 2 min under ice-cold conditions. After centrifugation at 300 × *g* for 5 min, the supernatant was removed, and the cells were washed twice with ice-cold PBS (1 mL × 2). The radioactivities of the precipitate and supernatant were measured using an auto-well gamma counter (Wizard 3, PerkinElmer Japan, Yokohama, Japan).

### Extracellular release study

As described above, C6 glioma cells were incubated with [^211^At]At-NpGT (30 kBq), [^125^I]I-NpGT (3.7 kBq), or [^18^F]F-NpGT (67 kBq) for 10 min, then 1000 μL of ice-cold PBS was added subsequently. After centrifuging the mixture at 300 × *g* for 5 min, the supernatant was discarded, and the cells were washed twice with 1000 μL of ice-cold PBS. Cells were resuspended in 10 mM HEPES/10% FBS/RPMI1640 medium (500 μL) and incubated at 37°C for 1, 10, or 30 min. The reaction was stopped by adding 1000 µL of ice-cold PBS, and the mixture was allowed to stand for 2 min under ice-cold conditions. After centrifugation at 300 × *g* for 5 min, the supernatant was removed, and the cells were washed twice with ice-cold PBS (1 mL × 2). The radioactivities of the precipitate and supernatant were measured using an auto-well gamma counter. In the study of [^125^I]I-NpGT, the supernatant was analyzed by RP-HPLC (System A).

### Inhibition assay

C6 glioma cells were suspended in 10 mM HEPES/HBSS (500 μL) containing 1 mM various inhibitors (L-tyrosine (Tyr), 2-aminobicyclo[2.2.1]heptane-2-carboxylic acid (BCH) (Sigma-Aldrich Japan, Tokyo, Japan), α-(methylamino)isobutyric acid (MeAIB) (Tokyo Chemical Industry Co., Ltd, Tokyo, Japan), α-methyl-L-tyrosine (AMT)) (Sigma-Aldrich Japan), and preincubate at 37°C for 5 min. Subsequently, [^211^At]At-NpGT (30 kBq), [^125^I]I-NpGT (3.7 kBq), or [^18^F]F-NpGT (35 kBq) dissolved in 10 mM HEPES/HBSS (20 μL) was added to the mixture and incubated for 30 min. The reaction was stopped by adding 1000 μL of ice-cold PBS. The reaction mixture was allowed to stand on ice for 2 min and centrifuged at 300 × *g* for 5 min. After centrifugation at 300 × *g* for 5 min, the supernatant was removed, and the cells were washed twice with ice-cold PBS (1 mL × 2). The radioactivities of the precipitate and supernatant were measured using an auto-well gamma counter.

### Stability against dehalogenation in PBS or FBS

[^211^At]At-NpGT (44.4 kBq), [^125^I]I-NpGT (11.1 kBq), or [^18^F]F-NpGT (1.0 MBq) dissolved in aqueous solution (5 μL) were added to either PBS (50 μL) or FBS, 50 μL). In the PBS stability studies, RP-TLC was performed at 1, 3, and 6 h for [^125^I]I-NpGT, and at 1 and 3 h for [^211^At]At-NpGT and [^18^F]F-NpGT to evaluate the percentage of the free halogen fraction. For stability studies in FBS with [^125^I]I-NpGT, after incubation for 24 h, acetonitrile (100 µL) was added to the reaction, and the contents were centrifuged at 13,000 rpm for 10 min. The supernatant was analyzed by RP-HPLC (System A) to determine the percentage of radioactivity corresponding to the free halogen fraction. In the other studies performed [^211^At]At-NpGT or [^18^F]F-NpGT, after incubation for 3 h, acetonitrile (100 µL) was added to the reaction mixture, and the procedure above for sample analysis was repeated to determine the percentage of radioactivity corresponding to the free halogen fraction.

### Preparation of animals

Animal studies were conducted in accordance with the institutional guidelines approved by the Chiba University Animal Care Committee or Osaka University Animal Care Committee. Six-week-old male ICR normal mice and five-week-old male BALB/c nude mice were purchased from Japan SLC, Inc. (Hamamatsu, Japan). C6 glioma cells (5.0 × 10^6^ cells) were suspended in 100 μL of culture medium and Matrigel (1:1 ratio; BD Biosciences, Franklin Lakes, NJ, USA). Tumor xenograft models were prepared by subcutaneously injecting a suspension of C6 glioma cells into immunodeficient nude mice.

### Biodistribution of tumor-bearing mice

The animal study was conducted according to the protocol reviewed and approved by Chiba University Animal Care Committee (Permit No. 4–183) or Osaka University Animal Care Committee (Permit No. 30–103-008). One week after C6 glioma cell transplantation, nude mice were injected intravenously with [^211^At]At-NpGT (30 kBq/mouse), [^125^I]I-NpGT (7.4 kBq/mouse), [^18^F]F-NpGT(400 kBq/mouse) or [^125^I]IMT (7.4 kBq/mouse) in PBS (100 μL). At 1 and 3 h post-injection., the mice (*n* = 4–5) were euthanized by cervical dislocation after isoflurane (Viatris Inc., New York) inhalation, and the organs were dissected. The organs of interest were weighed, and radioactivity counts were measured using an auto-well gamma counter.

#### Urine analysis

Male ICR mouse was injected intravenously with [^125^I]I-NpGT (185 kBq/mouse) in PBS (100 μL). At 6 h post-injection, a urine sample was collected. The urine sample (100 μL) was filtered through a 10 kDa cutoff ultrafiltration membrane (Sartorius, Germany) before being analyzed by RP-HPLC (system D).

### Therapeutic study

The animal study was conducted according to the protocol reviewed and approved by Chiba University Animal Care Committee (Permit No. 5–225). Tumor-bearing mice were prepared in the same manner as described in the biodistribution study. After tumor volumes had reached approximately 150–200 mm^3^, [^211^At]At-NpGT (0.1 MBq/mouse (n = 5), 0.3 MBq/mouse (n = 5)) or D-PBS(-) (control (n = 5)) was administered intravenously. Tumor size (mm^3^) was measured using calipers and calculated using the following elliptical sphere model equation:

$${\text{V }} = { 4}/{3 } \times \, \pi \, \times {\text{ a}}^{{2}} \times {\text{ b}}$$

where V is the volume of the tumor (mm^3^), a is the shorter radius (mm), and b is the longer radius (mm)).

In case of weight loss of more than 20%, the appearance of moribund state signs, or tumor size greater than 800 mm^3^, the mice were euthanized humanely by isoflurane inhalation.

### Statical analysis

All data are presented as the mean ± standard deviation (SD) of at least three independent measurements. Biodistribution studies were analyzed using one-way analysis of variance (ANOVA) followed by Tukey’s test for multiple comparisons (GraphPad Prism; GraphPad Software, San Diego, CA, USA). Statistical significance was set at P < 0.05.

## Results

### Synthesis and radiolabeling

The synthesis procedures for [^211^At]At-NpGT, [^125^I]I-NpGT, and [^18^F]F-NpGT are depicted in Additional file 1: Scheme S1. [^211^At]At-NpGT and [^125^I]I-NpGT were obtained with radiochemical yields of 44.3% (in 2 steps) and 40.9% (in 2 steps), respectively, by reaction with precursor **4** at 37 ˚C. In contrast, [^18^F]F-NpGT was obtained with a radiochemical yield of 35.4% (in 2 steps) by reaction with precursor **4** at 100 ˚C. Both [^125^I]-NpGT and [^18^F]-F-NpGT showed single peaks at retention times identical to those of non-radioactive compounds on RP-HPLC (Additional file 1: Fig. S1). For ^211^At, a non-radioactive iodine-labeled amino acid derivative (I-NpGT) was used as an authentic sample because there were no non-radioactive astatine isotopes. [^211^At]At-NpGT exhibited a single peak with a retention time similar to that of I-NpGT (Additional file 1: Fig. S1). The radiochemical purities of all compounds determined by RP-HPLC were greater than 99%, and subsequent experiments were performed using these radiolabeled compounds.

### Stability study

All radiolabeled compounds showed high stability in PBS and FBS, and no halogen desorption was observed (< 1%) (Table 1).

**Table 1:** Stability of [^211^At]At-NpGT, [^125^I]I-NpGT, and [^18^F]F-NpGT in PBS and FBS

	% of free halogen		
In PBS*	1 h	3 h	6 h
[^211^At]At-NpGT	< 1%	< 1%	N/A
[^125^I]I-NpGT	< 1%	< 1%	< 1%
[^18^F]F-NpGT	< 1%	< 1%	N/A

In FBS**	1 h	3 h	6 h
[^211^At]At-NpGT	< 1%	< 1%	N/A
[^125^I]I-NpGT	< 1%	< 1%	< 1%
[^18^F]F-NpGT	< 1%	< 1%	N/A

^*^The data was performed by TLC

^**^The data was performed by RP-HPLC

### In vitro experiments

**Fig. 2:**
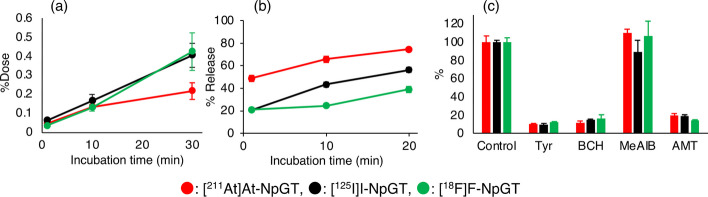
Cellular uptake and release of [^211^At]At-NpGT, [^125^I]I-NpGT and [^18^F]F-NpGT from C6 cells. **a** Time-course study of uptake. **b** Time-course study of extracellular release. The Y-axis shows the percentage release of each agent. **c** Inhibition of each agent’s uptake with amino acids and a LAT1-selective inhibitor (1 mM). The Y-axis shows the percentage of the control. The inhibitors were as follows Tyr = L-tyrosine; BCH = 2-aminobicyclo-(2,2,1)-heptane-2-carboxylic acid; MeAIB = α-methyl-aminoisobutyric acid; AMT = α-methyl-L-tyrosine

Cellular uptake of [^211^At]At-NpGT, [^125^I]I-NpGT, and [^18^F]F-NpGT by C6 glioma cells increased over time (Fig. 2a). Furthermore, these radiolabeled compounds were gradually excreted from the cells (Fig. 2b). RP-HPLC analysis revealed that the intact [^125^I]I-NpGT was excreted from the cells (Additional file 1: Fig. S2). The uptake of these radiolabeled compounds was significantly inhibited in the presence of BCH, an inhibitor of the L-type amino acid transporter, AMT, a LAT1-recognizing amino acid, and L-tyrosine (Fig. 2c). In contrast, MeAIB, an A-type amino acid transporter inhibitor, did not inhibit the cellular uptake.

### Biodistribution study

**Fig. 3:**
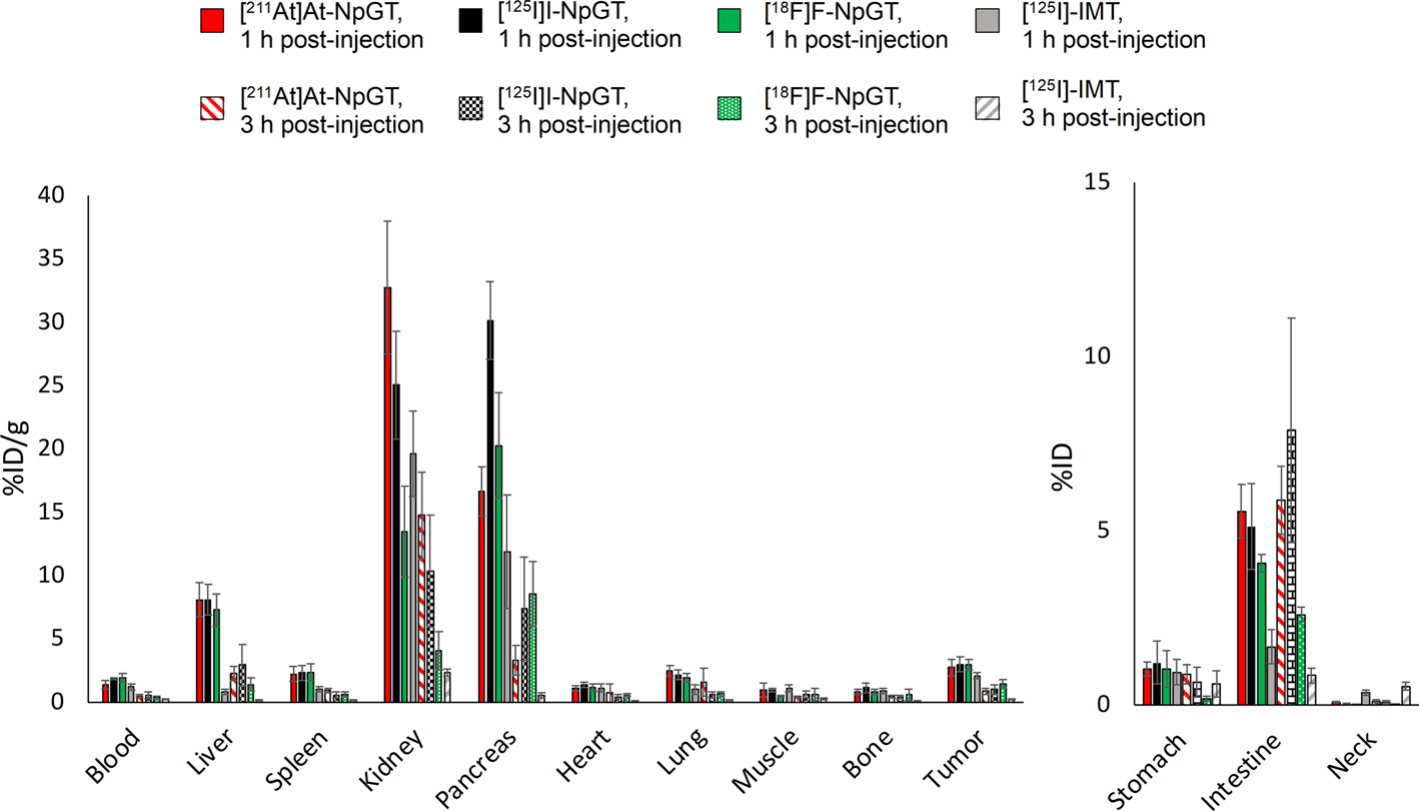
Biodistribution of [^211^At]At-NpGT, [^125^I]I-NpGT, [^18^F]F-NpGT and [^125^I]-IMT at 1 h and 3 h Post-injection in C6 bearing mice. Activity uptake in distinct organs is expressed as percentage of injected dose per organ mass (%ID/g) or as percentage of injected dose (%ID)

[^211^At]At-NpGT, [^125^I]I-NpGT, and [^18^F]F-NpGT were administered to tumor-bearing mice, and their pharmacokinetics were evaluated (Fig. 3, Table 2). We used [^125^I]-IMT as a reference radiolabeled amino acid tracer, the ^123^I-labeled form of which has been reported to be a cancer-diagnostic agent. [^211^At]At-NpGT, [^125^I]I-NpGT, and [^18^F]F-NpGT showed rapid blood clearance. Both [^211^At]At-NpGT and [^125^I]I-NpGT showed low accumulation in the stomach and thyroid glands. The accumulation of [^18^F]F-NpGT in the bone was low. [^211^At]At-NpGT, [^125^I]I-NpGT, and [^18^F]F-NpGT showed similar biodistribution in all tissues at both 1 and 3 h post-injection. In contrast, [^211^At]At-NpGT, [^125^I]I-NpGT, and [^18^F]F-NpGT showed higher tumor accumulation than [^125^I]-IMT and significantly higher tumor retention, even 3 h post-injection. The tumor-to-blood ratios of these radiolabeled compounds were higher than those of [^125^I]-IMT at 3 h post-injection.

**Table 2:** Biodistribution at 1 h and 3 h Post-injection in Tumor-Bearing Mice^a^

	[^211^At]At-NpGT		[^125^I]I-NpGT		[^18^F]F-NpGT		[^125^I]I-IMT	
Time after Injection	1 h	3 h	1 h	3 h	1 h	3 h	1 h	3 h
Blood	1.39 ± 0.33 ^c^	0.49 ± 0.09	1.86 ± 0.09	0.54 ± 0.27	1.91 ± 0.35	0.37 ± 0.10	1.22 ± 0.24^f,g^	0.22 ± 004^e,f^
Liver	8.09 ± 1.34	2.25 ± 0.55	8.09 ± 1.22	2.94 ± 1.63	7.28 ± 1.30	1.40 ± 0.53	0.80 ± 0.20^f,g^	0.14 ± 0.04^e,f^
Spleen	2.22 ± 0.58	0.94 ± 0.17^b^	2.30 ± 0.58	0.56 ± 0.24	2.36 ± 0.68	0.63 ± 0.20	1.01 ± 0.22^f,e,g^	0.16 ± 0.05^e,f,g^
Kidney	32.8 ± 5.23^c^	14.8 ± 3.41^c^	25.0 ± 4.25^d^	10.4 ± 4.42^d^	13.5 ± 3.58	4.06 ± 1.53	19.6 ± 3.37	2.34 ± 0.31^e,f^
Pancreas	16.6 ± 1.03^b^	3.33 ± 1.17	30.1 ± 3.05	7.36 ± 4.07	20.3 ± 4.20	8.54 ± 2.55	11.9 ± 4.48 ^f^	0.56 ± 0.21^f,g^
Heart	1.09 ± 0.20	0.74 ± 0.73	1.36 ± 0.20	0.43 ± 0.18	1.16 ± 0.30	0.56 ± 0.11	1.12 ± 0.29	0.11 ± 0.03
Lung	2.46 ± 0.41^c^	1.59 ± 1.10	2.14 ± 0.37^d^	0.58 ± 0.22	1.89 ± 0.35	0.65 ± 0.17	1.00 ± 0.39 ^e, f^	0.17 ± 0.03^e^
Muscle	0.95 ± 0.53	0.42 ± 0.07	0.96 ± 0.14	0.59 ± 0.27	0.48 ± 0.08	0.62 ± 0.48	1.10 ± 0.28	0.27 ± 0.09
Bone	0.81 ± 0.23	0.44 ± 0.12	1.14 ± 0.01	0.42 ± 0.16	0.83 ± 0.22	0.58 ± 0.42	0.87 ± 0.06	0.14 ± 0.02
Tumor	2.72 ± 0.63	0.86 ± 0.23^c^	2.97 ± 0.58	0.99 ± 0.37	2.95 ± 0.40	1.46 ± 0.33	2.07 ± 0.23 ^g^	0.25 ± 0.03^e,f,g^
Tumor/Blood	2.02 ± 0.51	1.73 ± 0.26^c^	1.59 ± 0.28	1.98 ± 0.39^d^	1.56 ± 0.15	3.96 ± 0.26	1.74 ± 0.35	1.17 ± 0.31^e,f,g^
Intestine*	5.56 ± 0.77^c^	5.88 ± 0.98^c^	5.12 ± 1.23	7.89 ± 3.22^d^	4.07 ± 0.25	2.60 ± 0.21	1.67 ± 0.49^e,f,g^	0.85 ± 0.21^f^
Stomach*	1.03 ± 0.20	0.88 ± 0.28^c^	1.22 ± 0.62	0.65 ± 0.43	1.03 ± 0.52	0.19 ± 0.07	0.94 ± 0.36	0.61 ± 0.38
Neck*	0.09 ± 0.02	0.11 ± 0.03	0.03 ± 0.01	0.09 ± 0.03	0.02 ± 0.01	0.01 ± 0.00	0.35 ± 0.06^e,f,g^	0.54 ± 0.11^e,f,g^

^a^Tissue radioactivity was expressed as %ID/g ± SD for each group (n = 5)

^*^Tissue radioactivity was expressed as %ID. Statistical analysis was performed using on-way ANOVA followed by Tukey’s test between [^211^At]At-NpGT and [^125^I]I-NpGT (^b^), [^211^At]At-NpGT and [^18^F]F-NpGT (^c^), [^125^I]I-NpGT and [^18^F]F-NpGT (^d^), [^211^At]At-NpGT and [^125^I]I-IMT (^e^), [^125^I]I-NpGT and [^125^I]-IMT (^f^), and [^18^F]F-NpGT and [^125^I]I-IMT (^g^)

### Urine analysis

The urine sample (100 μL) was filtered through a 10 kDa cutoff ultrafiltration membrane with a 73% recovery rate. RP-HPLC analysis revealed the presence of intact [^125^I]I-NpGT and unidentified metabolites in the urine (Additional file 1: Fig. S3). Very little radioactivity was observed in the void volume fraction where free iodine elutes.

### Therapeutic study

**Fig. 4:**
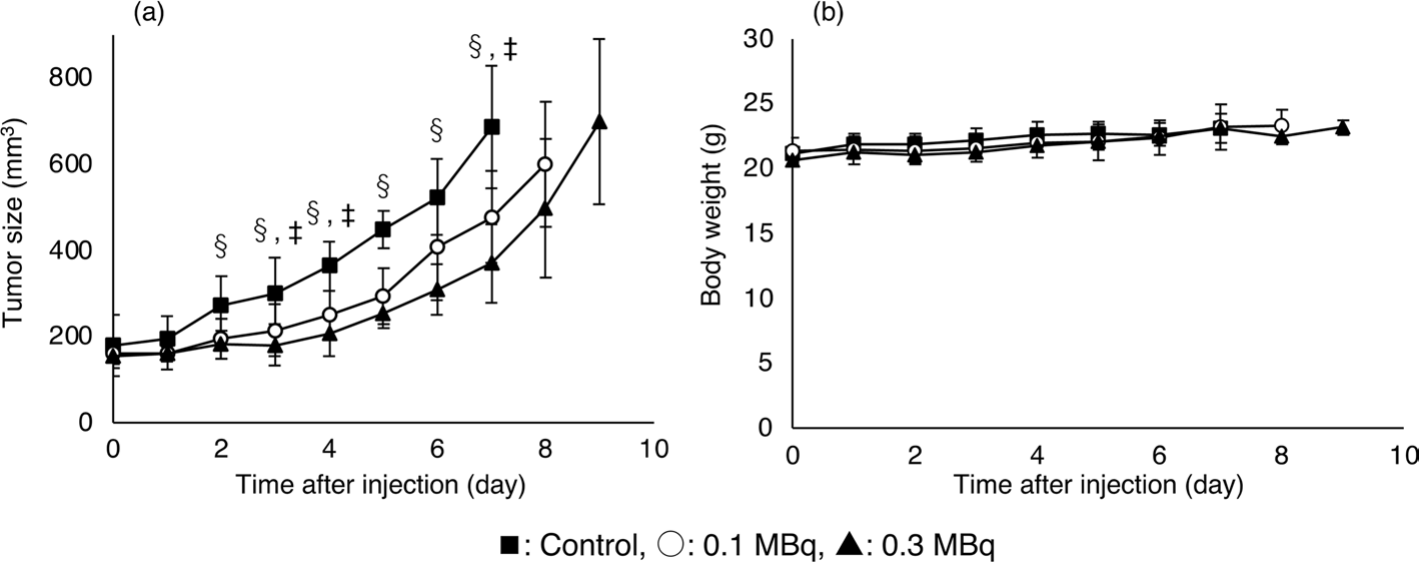
Tumor growth inhibition (**a**) and body weight change (**b**) by [^211^At]At-NpGT. Statistical significances were determined by Tukey’s test; *p* < 0.05 compared with 0.1 MBq (‡), 0.3 MBq(§)

Treatments were performed by administering [^211^At]At-NpGT (0.1 MBq/mouse or 0.3 MBq/mouse) or PBS (control) to C6 glioma-bearing mice. [^211^At]At-NpGT significantly inhibited the growth of C6 glioma tumors in a dose-dependent manner compared to control mice. (Fig. 4a). Even in the group that received 0.3 MBq/mouse [^211^At]At-NpGT, changes in body weight were not significantly different from those in control mice (Fig. 4b).

## Discussion

Radiohalogen encompass various isotopes, including ^18^F employed in PET diagnosis, ^123^I and ^131^I used in SPECT diagnosis, ^131^I used for β^−^-ray therapy, and ^211^At expected for α-ray therapy. The combination of these radionuclides holds promising potential for applications in radiotheranostics. Ensuring similarity in the pharmacokinetics of diagnostic and therapeutic agents in the body is essential for their application in radiotheranostics. While the stability of radioiodine- or ^18^F-labeled compounds containing benzene ring as a radiolabeling moiety is high, ^211^At-labeled compounds, especially ^211^At-labeled low-molecular-weight compounds, show low stability against deastatination in vivo, making the radiotheranostics use difficult (Watabe et al. 2020; Kaneda-Nakashima et al. 2021; Garg et al. 1990; Guerard et al. 2013). Although radiohalogens contain many useful radionuclides, it is challenging to employ them in radiotheranostics, especially when combining ^18^F and ^211^At. To address this issue, we previously showed that a neopentyl structure with two hydroxyl groups (NpG) can hold ^211^At and radioiodine stably in vivo (Suzuki et al. 2021). Additionally, ^18^F-labeled compounds containing NpG are stable in vivo (Shimizu et al. 2019). Based on these findings, we designed an NpG-conjugated L-tyrosine by introducing the NpG structure into the phenolic hydroxyl group of L-tyrosine. As shown in Table 2, accumulation of [^211^At]At-NpGT in the spleen, lungs, stomach, and thyroid was low. [^125^I]I-NpGT accumulation in the stomach and thyroid was low. The accumulation of [^18^F]F-NpGT in the bone was also low. These results indicate that NpG can effectively retain radiohalogens such as ^211^At, even when labeling low-molecular-weight compounds such as L-tyrosine. In addition, RP-HPLC analysis of urine sample showed that free iodine fractions (the void volume) had little radioactivity (Additional file 1: Fig. S3), also supporting high stability against dehalogenation in vivo.

In the present in vitro cell-based study (Fig. 2), [^211^At]At-NpGT, [^125^I]I-NpGT, and [^18^F]F-NpGT were taken up and released from the tumor cells, indicating that these radiolabeled compounds were co-transported. These characteristics are consistent with those of LAT1(Hafliger and Charles 2019). The cellular uptakes of [^211^At]At-NpGT, [^125^I]I-NpGT, and [^18^F]F-NpGT were similarly inhibited to the same extent by BCH, AMT, and L-Tyr, respectively (Fig. 2c). These results suggest that, although the calculated molecular weights of these compounds were significantly different ([^211^At]At-NpGT:493, [^125^I]I-NpGT:407, and [^18^F]F-NpGT:300), these radiolabeled compounds were similarly recognized by LAT1. LAT1-targeting radiolabeled amino acid derivatives such as [^18^F]-FET and para-borono-L-phenylalanine (BPA) have introduced substitutions at the phenolic hydroxyl group or para-position of the benzene ring (Fig. 1). Consequently, the modification of the phenolic hydroxyl group is expected to have minimal impact on LAT1 recognition. The present NpG-radiolabeled compounds were also modified on the hydroxyl group of the phenol of L-tyrosine (para-position of the benzene ring). Similar recognition of LAT1 for these radiolabeled compounds may indicate that, despite significant variations in their molecular weights, the molecular sizes of these compounds are not substantially different.

In biodistribution studies, [^211^At]At-NpGT, [^125^I]I-NpGT, and [^18^F]F-NpGT were highly accumulated in the tumor and were only moderately retained (Table 2). As LAT1 is an amino acid exchanger, the accumulation of these radiolabeled compounds in tumors gradually decreases with blood clearance. Nonetheless, the rapid clearance of these radiolabeled compounds from the bloodstream has enabled the attainment of high tumor-to-blood ratios, thereby offering valuable properties for the development of radiopharmaceuticals. [^211^At]At-NpGT, [^125^I]I-NpGT, and [^18^F]F-NpGT were highly distributed in the kidney and pancreas immediately after injection and were rapidly excreted from these organs. These biodistribution patterns are similar to those of other radiolabeled amino acid derivatives (Heiss et al. 1999; Shikano et al. 2004). However, it should be noted that radiolabeled amino acid derivatives such as [^18^F]-FET and [^123^I]-IMT have been shown to accumulate in the pancreas in mouse studies but not in humans (Jager et al. 1998; Pauleit et al. 2003). While the reason underlying these differences remains unknown, some studies have suggested differences in the expression patterns of LAT1 in the pancreas of mice and humans as a possible cause (Rooman et al. 2013; Nakada et al. 2014).

[^211^At]At-NpGT, [^125^I]I-NpGT, and [^18^F]F-NpGT showed higher accumulation in the liver and intestines than [^125^I]-IMT. This accumulation could be due to the increased lipophilicity of the radiolabeled compounds. Since accumulation in the liver has also been observed for [^18^F]-FMT, this observation in our compounds was thought to be due to the introduction of substituents to the hydroxyl group of phenol in L-tyrosine (Kaira et al. 2007). As no nonspecific accumulation in the abdomen has been observed for [^18^F]-FMT in clinical studies, we believe that the amount of radiolabeled NpG-conjugated tyrosine derivatives that accumulated in the liver and intestine in this study would not be a significant concern when used in clinical practice. In the mouse model used to evaluate the therapeutic effect, tumor cells proliferated rapidly, exceeding 800 mm^3^ by day 8 of the experiment. Despite this rapid tumor growth, the 0.1 MBq/mouse dose of [^211^At]At-NpGT significantly inhibited tumor growth. Furthermore, the 0.3 MBq/mouse dose group showed high tumor growth inhibition and no weight loss. These results indicated that [^211^At]At-NpGT was highly effective as an α-ray therapeutic agent for cancer.

Radionuclide-labeled amino acid derivatives targeting LAT1 initially accumulated in tumor cells but were gradually excreted. This is because LAT1 is a co-transported amino acid transporter, and radiolabeled amino acid derivatives, such as [^18^F]-FET and [^123^I]-IMT, are not used for protein synthesis (Wester et al. 1999; Lahoutte et al. 2001). Although radiometabolites in tumor cells were not evaluated in this study, considering previous reports on amino acid derivatives, it is likely that [^211^At]At-NpGT, [^125^I]I-NpGT, and [^18^F]F-NpGT were not used for protein synthesis and, as a result, their gradual excretion from the tumor may have been observed. When the radioactivity excreted from tumor cells incorporating [^125^I]I-NpGT was analyzed by RP-HPLC, the main radioactivity was derived from intact [^125^I]I-NpGT (Additional file 1: Fig. S2). These results suggested that [^125^I]I-NpGT was co-transported into and out of tumor cells. The excretion of radioactivity in tumors is unsuitable for radiotherapy. However, due to the short half-life of ^211^At (7.2 h), it is possible that ^211^At can efficiently irradiate tumor cells even during its short residence time. Based on these considerations, ^211^At-labeled amino acid derivatives, including [^211^At]At-NpGT, are considered to have high therapeutic efficacy. Furthermore, since the [^211^At]At-NpGT produced in this study did not show dehalogenation in vivo, [^211^At]At-NpGT is expected to have a higher therapeutic effect than the other ^211^At-labeled amino acid derivatives, notwithstanding the absence of comparative data.

In this study, [^211^At]At-NpGT, [^125^I]I-NpGT, and [^18^F]F-NpGT were prepared using triflate precursor compound 4. These radiolabeled compounds were obtained from compound 4 in a 2-step reaction with 30–40% radiochemical yield. The radiochemical conversion rates in each reaction were over 80%; however, the radioactivity decreased because of Sep-Pak and HPLC purification, resulting in low radiochemical yields. While the present study focused on the biodistribution of radiolabeled compounds and the therapeutic effect of [^211^At]At-NpGT, we did not optimize the radiolabeling procedures. Therefore, further studies on optimization of radiolabeling procedures are required to obtain high radiochemical yields.

## Conclusion

[^211^At]At-NpGT, [^125^I]I-NpGT, and [^18^F]F-NpGT were synthesized by incorporating an NpG structure into the phenolic hydroxyl group of L-tyrosine. These radiolabeled compounds were recognized as substrates of LAT1 and showed similar biodistribution in tumor-bearing mice. In particular, the similarity in the biodistribution of [^211^At]At-NpGT and [^18^F]F-NpGT indicates that this pair of radiolabeled compounds would be useful for radiotheranostics. To the best of our knowledge, this is the first report on the similarity in the biodistribution of ^18^F-labeled and ^211^At-labeled compounds based on low-molecular-weight compounds. In this study, [^211^At]At-NpGT exhibited a dose-dependent inhibitory effect on the growth of glioma-bearing mice. These findings suggest that radiotheranostics holds promise with the use of [^18^F]F-NpGT and [^123/131^I]I-NpGT for diagnostic applications and [^211^At]At-NpGT and [^131^I]I-NpGT for therapeutic applications.

### Supplementary Information

**Additional file 1.** Supplementary material.

## Data Availability

Data and materials are available from the corresponding authors upon reasonable request.

## Abbreviations

%ID: Percentage of injected dose
%ID/g: Percentage of injected dose per organ mass
AMT: α-Methyl-L-tyrosine
ANOVA: Analysis of variance
BCH: 2-Aminobicyclo-(2,2,1)-heptane-2-carboxylic acid
BPA: Para-Borono-L-phenylalanine
FBS: Fetal bovine serum
FET: Fluoroethyl-L-tyrosine
FMT: 3-Fluoro-α-methyl L-tyrosine
HBSS: HEPES/Hanks’ balanced salt solution
HEPES: 4-(2-Hydroxyethyl)-1-piperazineethanesulfonic acid
IMT: 3-Iodo-α-methyl L-tyrosine
IPA: 4-Iodo-L-phenylalanine
LAT1: L-Type amino acid transporter 1
MeAIB: α-(Methylamino)isobutyric acid
NpG: Neopentyl glycol
PBS: Phosphate-buffered saline
PET: Positron emission tomography
P450: Cytochrome P450
RP-HPLC: Reverse-phase high-performance liquid chromatography
SD: Standard deviation
SPECT: Single-photon emission computed tomography
TLC: Thin layer chromatography
