# Supplementary material for: Neopentyl glycol-based radiohalogen-labeled amino acid derivatives for cancer radiotheranostics
Source: EJNMMI Radiopharm Chem. 2024 Feb 26;9:17. doi: 10.1186/s41181-024-00244-4 (PMC10897087; doi:10.1186/s41181-024-00244-4)
Supplement: Supplementary file 1 — Additional file 1. Supplementary material. [file 41181_2024_244_MOESM1_ESM.pdf]

Neopentyl Glycol-Based Radiohalogen-labeled Amino Acid Derivatives for Cancer Radiotheranostics

Yuta Kaizuka[1], Hiroyuki Suzuki[1]*, Tadashi Watabe[2,3], Kazuhiro Ooe[3], Atsushi Toyoshima[3,4], Kazuhiro Takahashi[5], Koichi Sawada[6], Takashi Iimori[6], Yoshitada Masuda[6], Takashi Uno[6,7], Kento Kannaka[1], Tomoya Uehara[1]*

Table of Contents

General. **4**

HPLC or TLC methods **4**

Synthesis **5**

2,2-Dimethyl-1,3-dioxane-5,5-dimethanol (2) *5*

2,2-Dimethyl-1,3-dioxane-5,5-diyl)bis(methylene) bis(trifluoromethanesulfonate) (3) *5*

N^{α}-(tert-butoxycarbonyl)-O-((2,2-dimethyl-5-((((trifluoromethyl)sulfonyl)oxy)methyl)-1,3-dioxan-5-yl)methyl)-L-tyrosine tert-butyl ester (4) *6*

N^{α}-(tert-butoxycarbonyl)-O-((5-(iodomethyl)-2,2-dimethyl-1,3-dioxan-5-yl)methyl)-L-tyrosine tert-butyl ester (5) *7*

O-(3-hydroxy-2-(hydroxymethyl)-2-(iodomethyl)propyl)-L-tyrosine (I-NpGT) *7*

N^{α}-(tert-butoxycarbonyl)-O-((5-(fluoromethyl)-2,2-dimethyl-1,3-dioxan-5-yl)methyl)-L-tyrosine tert-butyl ester (6) *8*

O-(3-hydroxy-2-(hydroxymethyl)-2-(fluoromethyl)propyl)-L-tyrosine (F-FNT) *8*

Radiosynthesis of [^{211}At]At-NpGT **9**

Radiosynthesis of [^{125}I]I-NpGT **9**

Radiosynthesis of [^{18}F]F-NpGT **9**

Radiosynthesis of [^{125}I]-IMT **10**

SCHEME S1. Synthetic scheme of At-NpGT, I-NpGT and F-NpGT **11**

FIGURE S1. RP-HPLC radiochromatograms of [^{211}At]At-NpGT, [^{125}I]I-NpGT, [^{18}F]F-NpGT and [^{125}I]-IMT. **12**

FIGURE S2. RP-HPLC radiochromatograms of [^{125}I]I-NpGT and radiometabolites released from C6 cells **13**

FIGURE S3. RP-HPLC radiochromatograms of [^{125}I]I-NpGT and radiometabolites in the urine. **14**

NMR characterization **15**

^{1}H-NMR and ^{13}C-NMR for compound 2 .... *15*

^{1}H-NMR and ^{13}C-NMR for compound 3 .... *16*

^{1}H-NMR and ^{13}C-NMR for compound 4 .... *17*

^{1}H-NMR and ^{13}C-NMR for compound 5 .... *18*

^{1}H-NMR and ^{13}C-NMR for I-NpGT *19*

^{1}H-NMR and ^{13}C-NMR for compound 6 .... *20*

^{1}H-NMR, ^{13}C-NMR and ^{19}F-NMR for F-NpGT *21*

Reference 23

General.

[^{125}I]NaI (ca. 3.7 MBq/μL) was purchased from PerkinElmer (Waltham, MA, USA). ^{211}At was supplied by the Research Center for Nuclear Physics at Osaka University through the Supply Platform of Short-lived Radioisotopes and from Fukushima Medical University. ^{211}At was produced by the ^{209}Bi(α, 2n)^{211}At reaction followed by separation and purification by a dry distillation method. ^{18}F was supplied by the Chiba University Hospital. ^{1}H-NMR, ^{13}C-NMR and ^{19}F-NMR spectra were recorded on a JEOL JNM-ECS 400 spectrometer (JEOL, Tokyo, Japan). Mass spectrometry was carried out using an AccuTOF LC-plus (JMS-T100LP, JEOL, Tokyo). HPLC purification was performed using a Hitachi L-2400 system coupled to a NaI(Tl) radioactivity detector (Gabi star, Raytest, Strubenhardt, Germany). The radioactivity counts were determined with an automated gamma well counter (Wizard 3, PerkinElmer Japan, Yokohama, Japan). *N*$^{\alpha}$-*tert*-butoxycarbonyl-L-tyrosine *tert-butyl ester* was prepared according to a previously described method (*1*). The other chemicals obtained from the commercial sources were of reagent grade or higher and were used without purification.

HPLC or TLC methods

Analytical reversed-phase HPLC (RP-HPLC) was performed with a Unison US-C18 column (4.6 × 150 mm, Imtakt, Kyoto, Japan) at a flow rate of 1 mL/min using a linear gradient mobile phase starting from 90% A (0.1% aqueous trifluoroacetic acid (TFA)) and 10% B (acetonitrile with 0.1% TFA) to 50% A and 5% B in 20 min, followed by 0% A and 100% B in 30 min (System A) or from 95% A and 5% B to 0% A and 100% B in 30 min (System B). In System C, the mobile phase was maintained 100% A and 0% B for 5 min, then changed to 70% A and 30% B, followed by a change 0% A and 100% B. In System D, the mobile phase was maintained 95% A (0.01 M acetate buffer (pH 6.0)) and 0% B (acetonitrile) for 5 min, then changed gradiently to 70% A and 30% B in 20 min, followed by a change 0% A and 100% B in 30 min. RP-TLC was performed using Silica gel 60 RP-18 F254S (Merck, Darmstadt, Germany) and was

developed in a mixture of tert-butyl alcohol : acetic acid : H_2O (4:1:1, v/v). Preparative RP-HPLC was performed with a Unison US-C18 column (20 × 150 mm, Imtact, Kyoto) at a flow rate of 5 mL/min using a linear gradient mobile phase starting from 90% A and 10% B to 50% A and 50% B in 30 min (System D).

Synthesis

***2,2-Dimethyl-1,3-dioxane-5,5-dimethanol* (2)**

Pentaerythritol (**1**) (6.00 g, 44.1 mmol) and (+)-10-camphorsulfonic acid (0.205 g, 0.881 mmol) were dissolved in *N,N*-dimethylformamide (DMF) (120 mL) and the solution was heated to 80 °C. After completion of dissolution, the temperature was reduced to 40 °C, and 2,2-dimethoxypropane (6.50 mL, 52.8 mmol) was added dropwise. The mixture was allowed to cool to room temperature and stirred continuously for 2 days. Triethylamine (370 µL, 2.65 mmol) was added to neutralize the reaction. After removing the solvent *in vacuo*, the residue underwent a Soxhlet extraction with hexane for 2 days. The solvent was removed *in vacuo*, then compound **2** was recrystallized from a mixture of ethyl acetate and hexane as a white solid (633 mg, 3.59 mmol, 36.1%). ^{1}H-NMR (DMSO-d_6): δ 1.29 (6H, s, CH_3), 3.35-3.36 (4H, d, CH_2), 3.59 (4H, s, CH_2), 4.48-4.51 (2H, t, OH). ^{13}C-NMR (DMSO-d_6): 23.83, 38.88, 60.50, 61.67, 91.12. HRMS(ESI) calcd. for $C_8H_{16}NaO_4$ [M + Na]+: m/z 199.0946, found 199.0940.

***2,2-Dimethyl-1,3-dioxane-5,5-diyl)bis(methylene) bis(trifluoromethanesulfonate)* (3)**

Compound **2** (633 mg, 3.59 mmol) was dissolved in dichloromethane (40 mL), and 2,6-lutidine (4.2 mL, 35.9 mmol) was added. After cooling to -78 °C, trifluoromethanesulfonic anhydride (Tf_2O) (2 mL, 12.2 mmol) was added dropwise, and then the solution was stirred for 1 h. The reaction temperature was subsequently raised to -20 °C and maintained overnight. The mixture was successively washed with saturated $NaHCO_3$ solution (20 mL), 5% citric acid solution (30 mL), and saturated brine (20 mL). The organic layer was dried over magnesium sulfate, and then the solvent

was evaporated *in vacuo*. The product was purified by silica gel chromatography using hexane: ethyl acetate (10:1) as an eluent to produce compound **3** as a white solid (1.30 g, 2.94 mmol, 82.0%). ^{1}H-NMR ($CDCl_3$): δ 1.44 (6H, s, CH_3), 3.79 (4H, s, CH_2), 4.57 (4H, s, CH_2). ^{13}C-NMR ($CDCl_3$): 23.39, 39.00, 60.88, 73.54, 99.71, 113.92, 117.11, 120.29, 123.47. HRMS(ESI) calcd. for $C_{10}H_{14}F_6NaO_8S_2$ $[M + Na]^+$: m/z 462.9932, found 462.9917.

<u>N^α-(tert-butoxycarbonyl)-O-((2,2-dimethyl-5-((((trifluoromethyl)sulfonyl)oxy)methyl)-1,3-dioxan-5-yl)methyl)-L-tyrosine tert-butyl ester (4)</u>

NaH (10.8 mg, 0.270 mmol) was suspended in tetrahydrofuran (THF) (0.50 mL). Under an argon atmosphere, a solution of compound **3** (76.0 mg, 0.226 mmol) in THF (1.50 mL) was added dropwise to the NaH suspension under ice-cold conditions. The mixture was stirred at room temperature for 30 min. Subsequently, compound (6) (100 mg, 0.226 mmol) was added, and the reaction mixture was stirred for 40 min at room temperature. After removing the solvent, the resulting residue was redissolved in ethyl acetate (10 mL) and washed thrice with a saturated $NaHCO_3$ solution (10 mL each). The organic phase was dried over magnesium sulfate. After removing the solvent, the residue was purified by a preparative TLC plate using a mixture of hexane: ethyl acetate (2:1) as a solvent to obtain compound **4** as a colorless oil (86.2 mg, 0.137 mmol, 60.8%). ^{1}H-NMR ($CDCl_3$): δ 1.41-1.45(24H, overlapped, CH_3), 2.99-3.01 (2H, t, CH_2), 3.81-3.93 (6H, overlapped, CH_2), 4.39-4.41 (1H, multiple, CH), 4.79 (2H, s, CH_2), 4.96-4.98 (1H, d, NH), 6.80-6.82 (2H, d, aromatic), 7.08-7.10 (2H, d, aromatic). ^{13}C-NMR ($CDCl_3$): 21.58, 25.62, 28.07, 28.43, 37.71, 38.90, 55.01, 61.85, 66.22, 75.43, 79.75, 82.16, 99.19, 113.94, 114.43, 117.12, 120.30, 123.48, 129.57, 130.61, 130.75, 155.19, 157.21, 170.93, 171.07. HRMS(ESI) calcd. for $C_{27}H_{40}F_3NaNNaO_{10}S$ [M + Na]+: m/z 650.2223, found 650.2218.

***N^{α}-(tert-butoxycarbonyl)-O-((5-(iodomethyl)-2,2-dimethyl-1,3-dioxan-5-yl)methyl)-L-tyrosine tert-butyl ester* (5)**

Compound **4** (64.6 mg, 0.103 mmol) was dissolved in acetonitrile (1 mL). After adding sodium iodide (46 mg, 0.309 mmol), the mixture was stirred overnight at room temperature. After removing the solvent, the resulting residue was redissolved in ethyl acetate (5 mL) and washed sequentially with a 5% $NaHCO_3$ solution (5 mL), twice with water (5 mL each), and finally with saturated brine (5 mL). The organic layer was dried over magnesium sulfate. After removing the solvent *in vacuo*, the residue was purified by a preparative TLC plate using a mixture of hexane: ethyl acetate (2:1) as an eluent to afford compound **5** as a colorless oil (51.4 mg, 0.0849 mmol, 82.4%). ^{1}H-NMR ($CDCl_3$): δ 1.42-1.44 (24H, overlapped, CH_3), 2.99-3.01(2H, t, CH_2), 3.41 (2H, s, CH_2), 3.78-3.91 (4H, multiple, CH_2), 3.98 (2H, s, CH_2), 4.40-4.41 (1H, multiple, CH), 4.95-4.97 (1H, d, NH), 6.83-6.85 (2H, d, aromatic), 7.07-7.09 (2H, d, aromatic). ^{13}C-NMR ($CDCl_3$): 10.42, 27.58, 24.74, 28.11, 28.45, 36.84, 37.63, 55.02, 64.84, 68.81, 79.73, 82.11, 98.85, 114.63, 129.00, 130.65, 155.21, 157.76, 171.11. HRMS(ESI) calcd for $C_{26}H_{40}INNaO_7$ [M + Na]+: m/z 628.1747, found 628.1778.

***O-(3-hydroxy-2-(hydroxymethyl)-2-(iodomethyl)propyl)-L-tyrosine* (I-NpGT)**

Compound (9) (10.2 mg, 16.8 nmol) was dissolved in a mixture of TFA (800 μL) and H_2O (200 μL), and the solution was stirred at room temperature for 5 h. After removing the solvent *in vacuo*, the residue w as subjected to azeotropic drying with acetonitrile (1 mL × 2). The residue was purified by preparative RP-HP LC (System D) to afford I-NpGT as a white solid (5.45 mg, 10.8 nmol, 64.1%). ^{1}H-NMR (D_2O): δ 2.95-3.14 (2H, multiple, CH_2), 3.20 (2H, s, CH_2), 3.51 (4H. s, CH_2), 3.79 (2H, s, CH_2), 4.04-4.07 (1H, q, CH), 6.86-6.8 8 (2H, d, aromatic), 7.07-7.09 (2H, d, aromatic). ^{13}C-NMR (D_2O): 9.39, 36.07, 44.10, 56.65, 61.77, 68.26, 115. 96, 128.24, 131.19, 158.65, 174.59. HRMS(ESI) calcd. for $C_{14}H_{20}INNaO_5$ [M + Na]+: m/z 432.0284, found 43 2.0314.

***N^α-(tert-butoxycarbonyl)-O-((5-(fluoromethyl)-2,2-dimethyl-1,3-dioxan-5-yl)methyl)-L-tyrosine tert-butyl ester* (6)**

Compound **4** (20.0 mg, 0.0319 mmol) was dissolved in THF (0.5 mL). To this solution, tetrabutylammonium fluoride (TBAF, approximately 1 mol/L in THF) (64 µL, 0.0637 mmol) was added. The mixture was then stirred at room temperature overnight. After removing the solvent, the residue was redissolved in ethyl acetate (5 mL) and successively washed with water twice (5 mL each), saturated ammonium chloride solution (5 mL), and saturated brine (5 mL). The organic layer was dried over magnesium sulfate. After removing the solvent *in vacuo*, compound **6** was obtained as a white solid (15.0 mg, 30.1 nmol, 94.5%).^{1}H-NMR ($CDCl_3$): δ 1.40-1.48 (24H, overlapped, CH_3), 2.96-3.00(2H, t, CH_2), 3.82-3.93 (6H, overlapped, CH_2), 3.98 (2H, s, CH_2), 4.37-4.39 (1H, multiple, CH), 4.52-4.63 (2H, d, CH_2) 4.92-4.94 (1H, d, NH), 6.80-6.82 (2H, d, aromatic), 7.04-7.07 (2H, d, aromatic). ^{13}C-NMR ($CDCl_3$): 23.23, 24.05, 28.00, 28.11, 28.33, 37.49, 39.00, 39.17, 54.91, 61.45 61.52, 66.69, 79.60, 81.88 ,81.99, 83.58, 98.54, 114.39, 128.92, 130.55, 155.09, 157.67, 170.97. HRMS(ESI) calcd. for $C_{26}H_{40}FNNaO_7$ [M + Na]+: m/z 520.2687, found 520.2691.

***O-(3-hydroxy-2-(hydroxymethyl)-2-(fluoromethyl)propyl)-L-tyrosine* (F-FNT)**

Compound **6** (12.0 mg, 20.4 nmol) was dissolved in a mixture of TFA (4.0 mL) and water (1.0 mL), and the resulting solution was stirred at 60 °C for 3 h. After removing the solvent, the residue was subjected to azeotropic drying using acetonitrile (1 mL × 2). The residue was purified by preparative RP-HPLC (System D) to afford F-FNT as a white solid (5.20 mg, 13.1 nmol, 64.0%) and was successfully isolated as a white solid. ^{1}H-NMR (D_2O): δ 2.99-3.16 (2H, multiple, CH_2), 3.59 (4H. s, CH_2), 3.91 (2H, s, CH_2), 4.02-4.04 (1H, q, CH), 4.46-4.54 (2H, d, CH_2), 6.91-6.92 (2H, d, aromatic), 7.13-7.14 (2H, d, aromatic). ^{13}C-NMR (D_2O): δ35.69, 46.18, 46.28, 55.62, 60.49, 60.53, 66.87, 83.37, 84.47, 115.98, 127.63, 131.27, 158.87, 173.19. ^{19}F-NMR (D_2O): -237.09, -237.03, -236.94. HRMS(ESI) calcd. for $C_{14}H_{20}FNNaO_5$ $[M + Na]^+$: m/z 324.1223, found 324.1214.

Radiosynthesis of [^{211}At]At-NpGT

^{211}At was dissolved in acetonitrile (20 μL), 10% sodium ascorbate solution (2.5 μL), and 1% *N, N*-diisopropylethylamine (DIEA)/acetonitrile solution of compound **4** (0.3 mg/50 μL) were added. The mixture was heated at 37°C for 30 min and passed through a Sep-Pak C18 cartridge using H_2O and acetonitrile as solvent. After removing the solvent, 80% trifluoroacetic acid (TFA)/H_2O was added to remove the protecting groups. The mixture was heated at 60°C for 30 min. TFA was evaporated by N_2 gas, and the solution was neutralized with a 2M sodium acetate solution. The solution was purified by RP-HPLC with system A, then desalted through a Sep-Pak C18 cartridge using H_2O and acetonitrile as solvents. The solvent was removed *in vacuo*, diluted with Phosphate-Buffered Saline (PBS), and used for further evaluation studies.

Radiosynthesis of [^{125}I]I-NpGT

A solution of [^{125}I]NaI (0.5 μL) was added to a 1% DIEA/acetonitrile solution of compound **4** (0.3 mg/50 μL). The mixture was heated at 37°C for 60 min and passed through a Sep-Pak C18 cartridge using H_2O and acetonitrile as solvent. After removing the solvent, 80% TFA/H_2O was added to remove the protecting groups. The mixture was heated at 60°C for 30 min. TFA was evaporated by N_2 gas, and the solution was neutralized with a 2M sodium acetate solution. The solution was purified by RP-HPLC with system A, then desalted through a Sep-Pak C18 cartridge using H_2O and acetonitrile as solvents. The solvent was removed *in vacuo*, diluted with PBS, and used for further evaluation studies.

Radiosynthesis of [^{18}F]F-NpGT

To the reaction vial, an aqueous potassium carbonate solution (5.85 mg/L, 0.3 mL), a Cryptofix 222 acetonitrile solution (14 mg/0.3 mL), and a solution containing ^{18}F were added. The mixture was concentrated using a CEV1B evaporator (BioChromato, Kanagawa, Japan) with heating to 100°C. Subsequently, super-dehydrated acetonitrile (Wako,

Osaka, Japan) was added to facilitate azeotropic evaporation. An acetonitrile solution of compound **4** (1.0 mg/50 μL) was added to the reaction vial. The mixture was heated at 100°C for 30 min and passed through a Sep-Pak C18 cartridge using H_2O and acetonitrile as solvent. After removing the solvent, 80% TFA/H_2O was added to remove the protecting groups. The mixture was heated at 60°C for 30 min. TFA was evaporated by N_2 gas, and the solution was neutralized with a 2M sodium acetate solution. The solution was purified by RP-HPLC with system C, then desalted through a Sep-Pak C18 cartridge using H_2O and acetonitrile as solvents. The solvent was removed *in vacuo*, diluted with PBS, and used for further evaluation studies.

Radiosynthesis of [^{125}I]-IMT

α-Methyl L-tyrosine (0.2 ng/35 μL in 0.4 M phosphate buffer (pH 6.2)) and [^{125}I]NaI (0.5 μL, ca. 1 MBq) were mixed. A 10 μL of Chloramine-T (0.45 mg/mL in 0.05 M phosphate buffer (pH 6.2)) was added to the mixture for 2 min at room temperature. The reaction was terminated by adding a 10% Na_2SO_4 solution (20 μL). The solution was purified by RP-HPLC using system B, and [^{125}I]I-IMT was desalted through a Sep-Pak C18 cartridge using H_2O and acetonitrile as solvent. The product solution was concentrated *in vacuo*, diluted with PBS, and used for further evaluation studies.

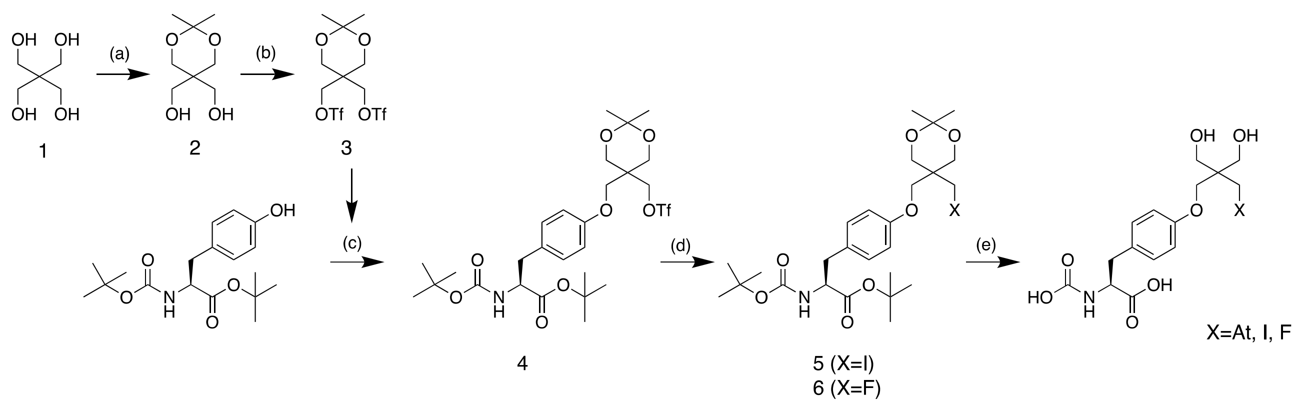

SCHEME S1. Synthetic scheme of At-NpGT, I-NpGT and F-NpGT

(a) (+)-10-camphorsulfonic acid, 2,2-dimethoxypropan, DMF, (b) 2,6-lutidine, Tf_2O, CH_2Cl_2, (c) NaH, THF, (d) NaI, DIPEA, acetonitrile, or At, DIPEA, ascrobic acid, acetonitrile, or TBAF, K_2CO_3, Cryptofix 222, acetonitrile, (e) 90%TFA/H_2O

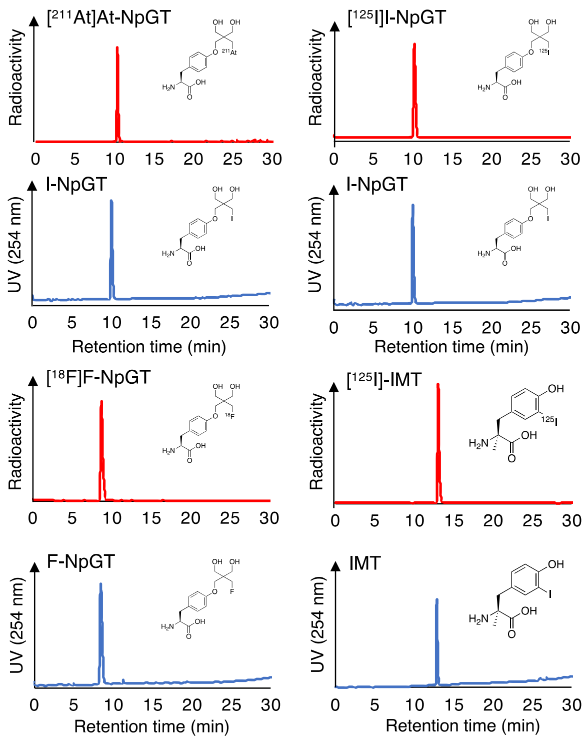

FIGURE S1. RP-HPLC radiochromatograms of [^{211}At]At-NpGT, [^{125}I]I-NpGT, [^{18}F]F-NpGT and [^{125}I]-IMT.

[^{125}I]I-NpGT, [^{18}F]F-NpGT and [^{125}I]-IMT showed similar retention times to their non-radioactive counterparts (UV chromatograms). In case of [^{211}At]At-NpGT, non-radioactive I-NpGT was used as an authentic sample because non-radioactive astatine isotopes were not present. System A was used for [^{211}At]At-NpGT, [^{125}I]I-NpGT and [^{18}F]F-NpGT analysis, and system B was used for [^{125}I]-IMT analysis.

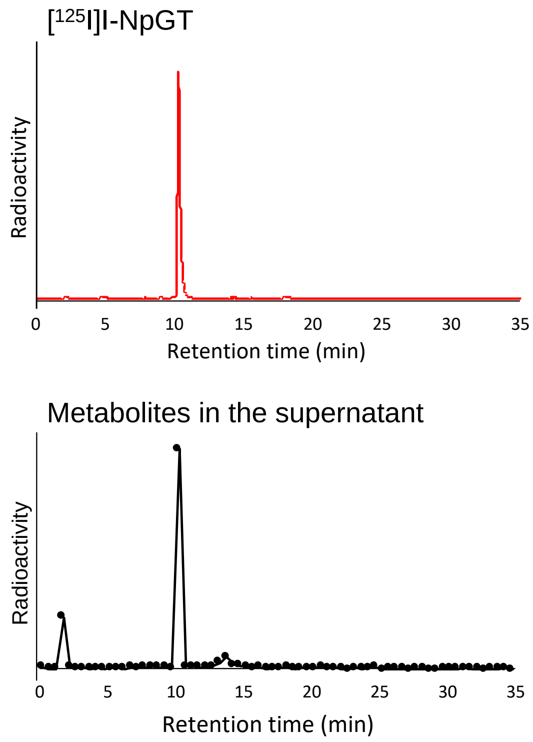

FIGURE S2. RP-HPLC radiochromatograms of [^{125}I]I-NpGT and radiometabolites released from C6 cells

In the extracellular release study of [^{125}I]I-NpGT, the supernatant was analyzed by RP-HPLC (System A). The presence of intact [^{125}I]I-NpGT indicates the involvement of LAT1, a co-transported amino acid transporter.

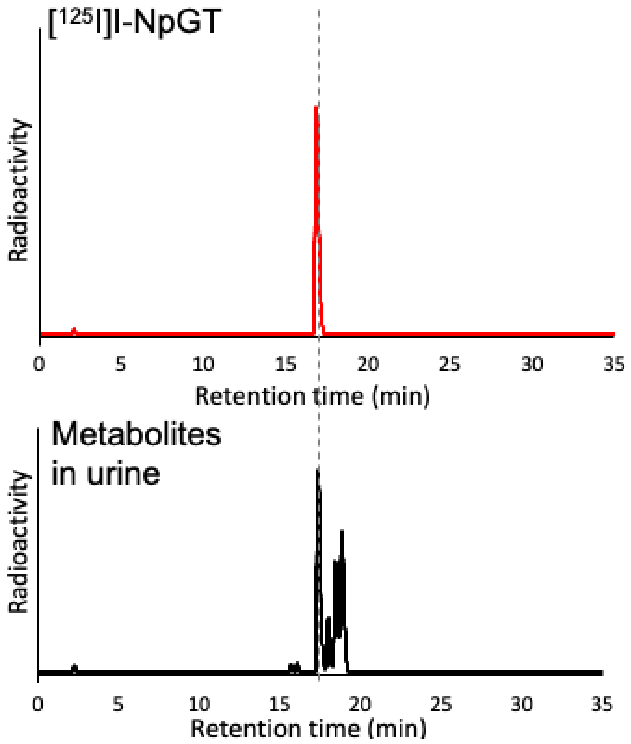

FIGURE S3. RP-HPLC radiochromatograms of [^{125}I]I-NpGT and radiometabolites in the urine.

[^{125}I]I-NpGT (185 kBq) was administered to ICR normal mice and urine was collected up to 6 hours after administration. The urine was analyzed using RP-HPLC (system D). As a result, we observed intact [^{125}I]I-NpGT and unidentified metabolites. Very little radioactivity was observed in the void volume fraction where free iodine elutes.

NMR characterization

^{1}H-NMR and ^{13}C-NMR for compound 2

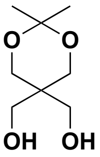

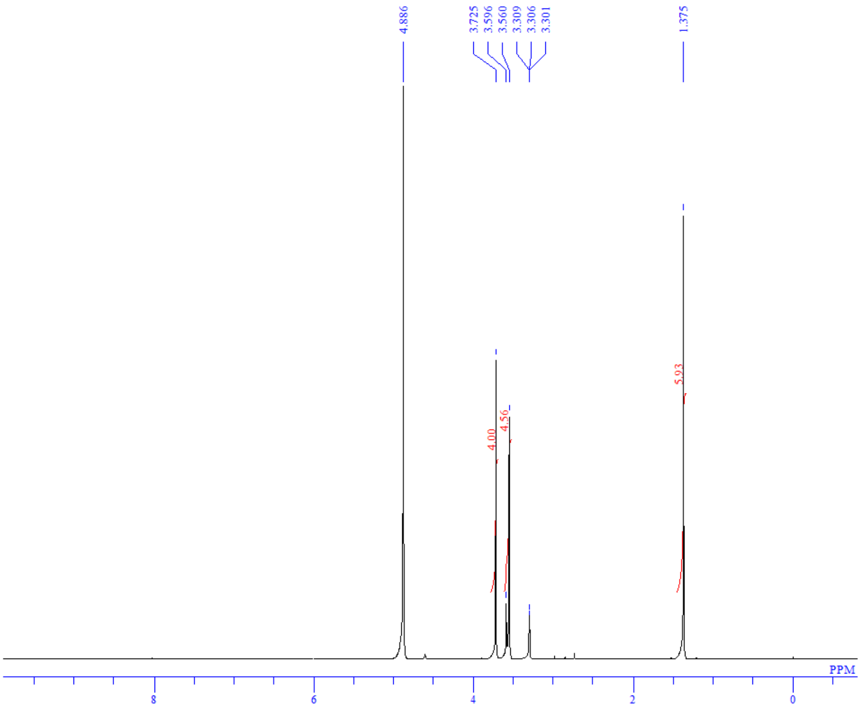

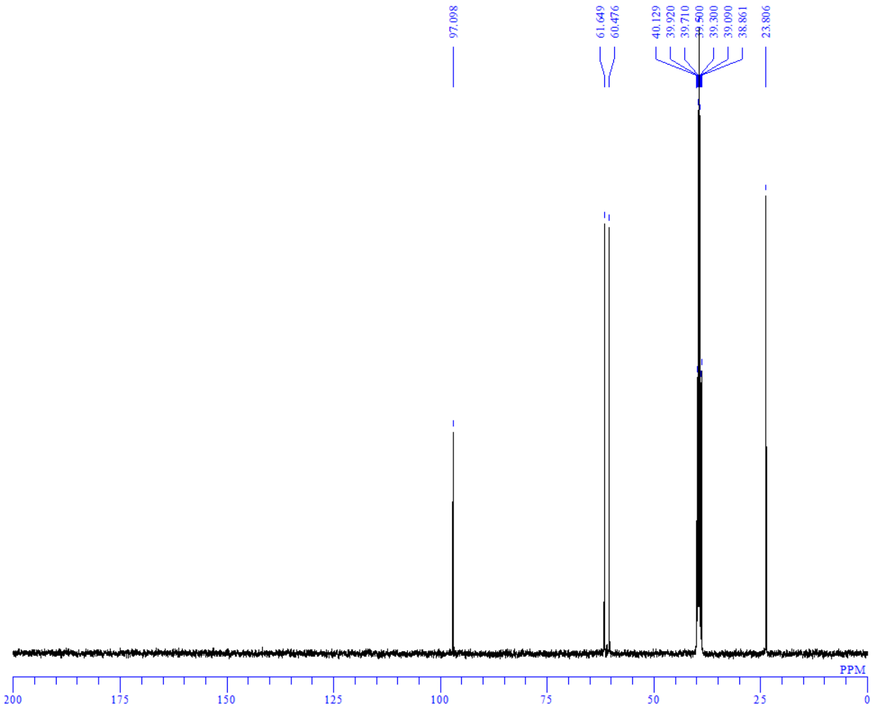

^{1}H-NMR and ^{13}C-NMR for compound 3

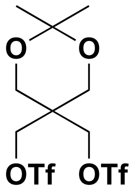

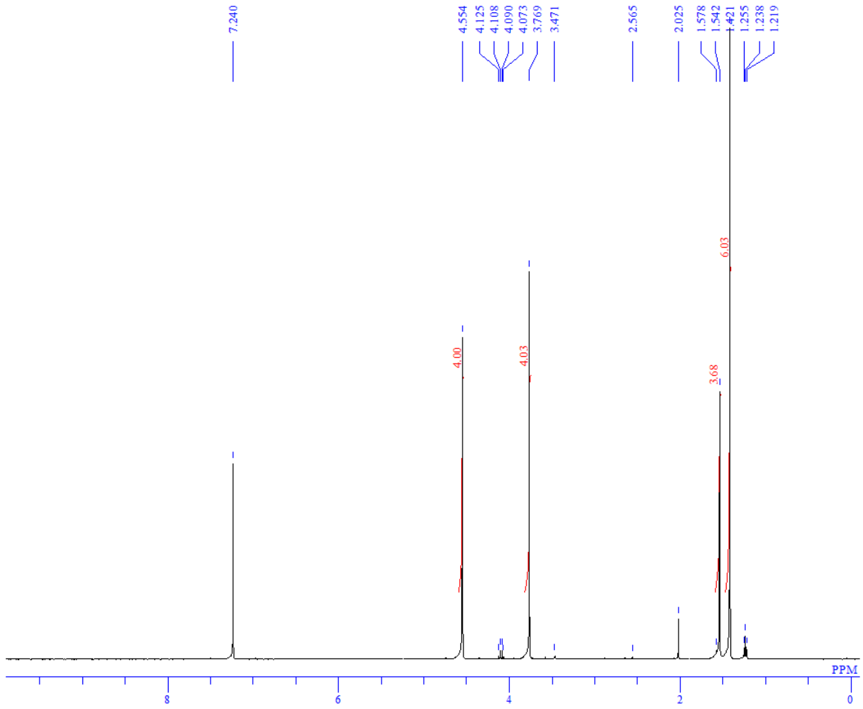

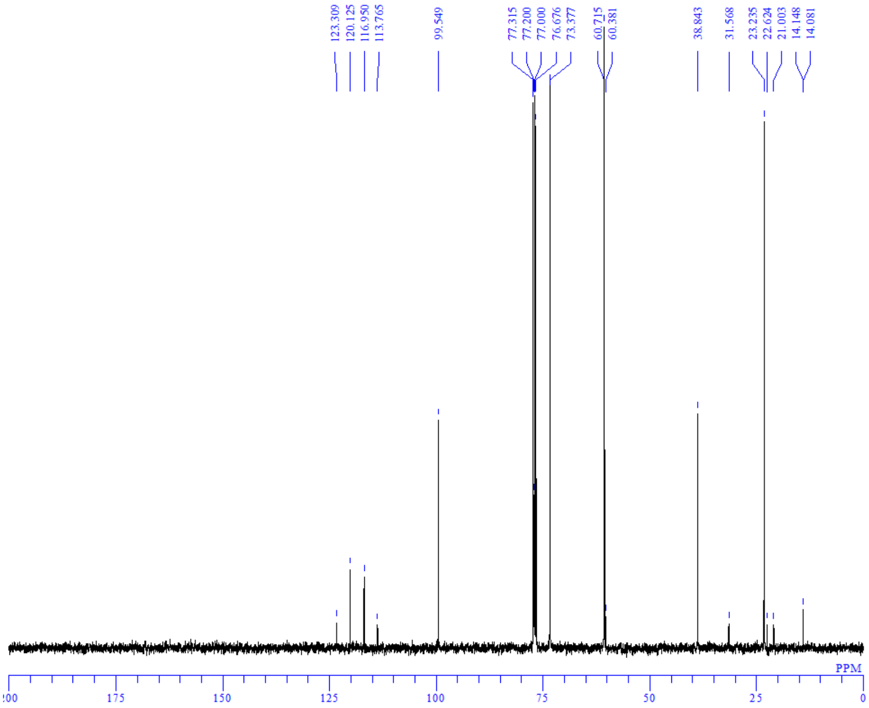

^{1}H-NMR and ^{13}C-NMR for compound 4

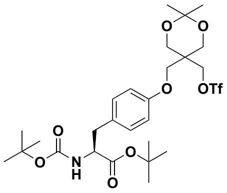

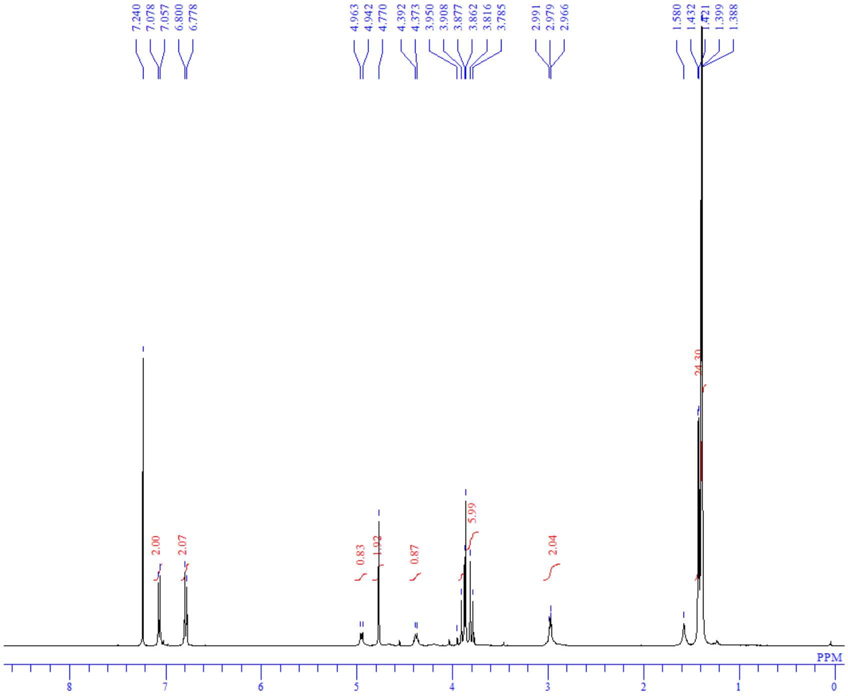

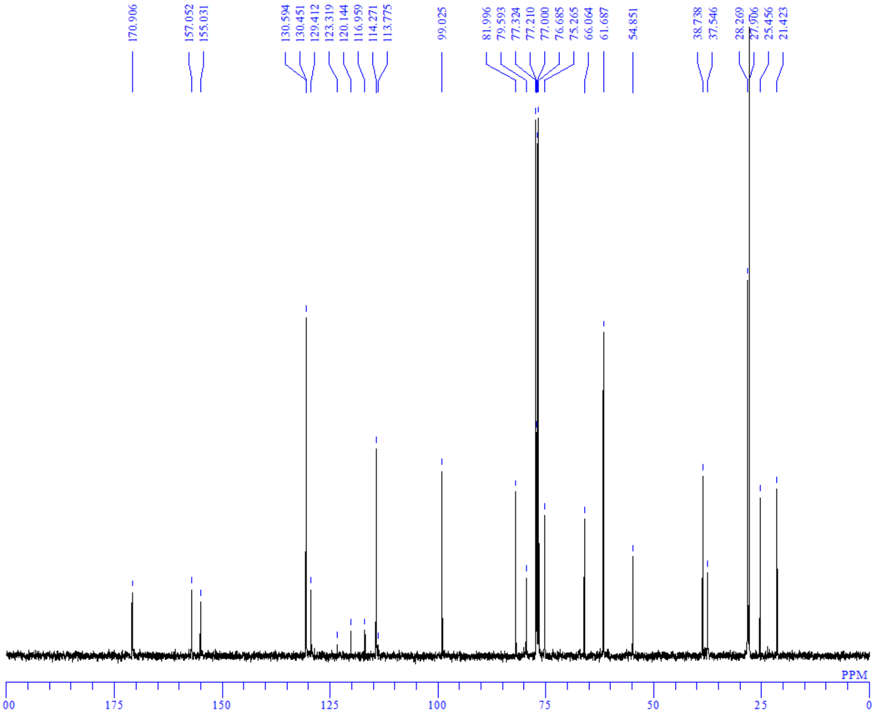

^{1}H-NMR and ^{13}C-NMR for compound 5

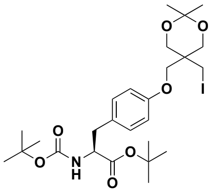

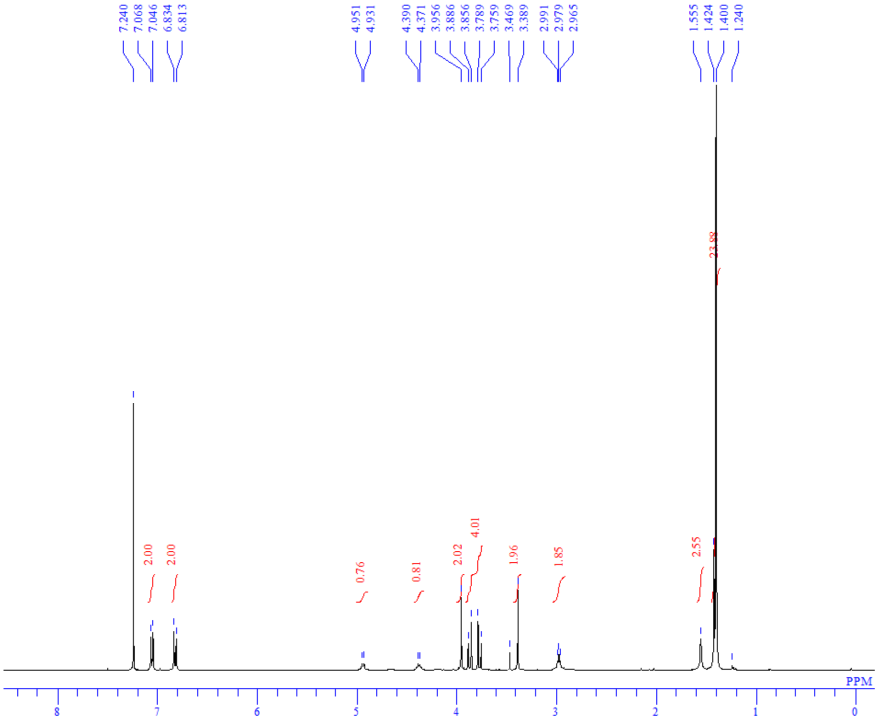

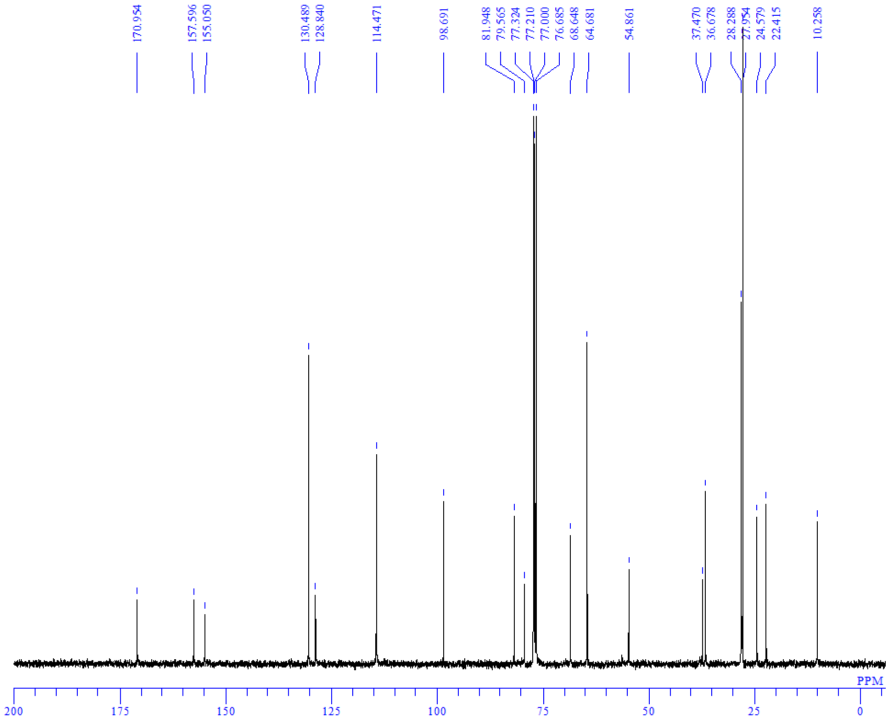

^{1}H-NMR and ^{13}C-NMR for I-NpGT

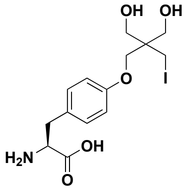

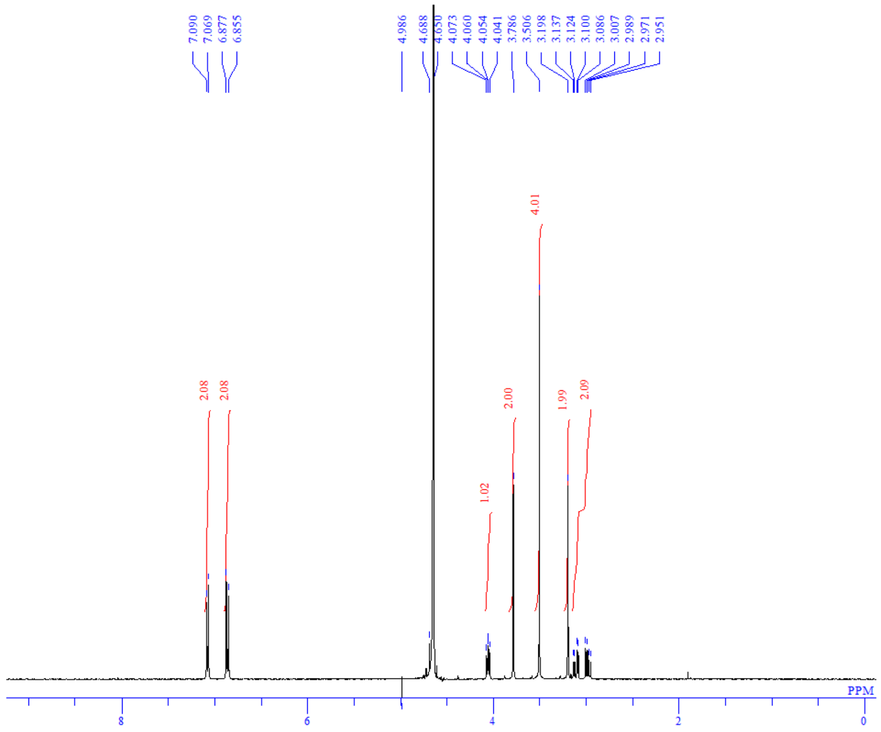

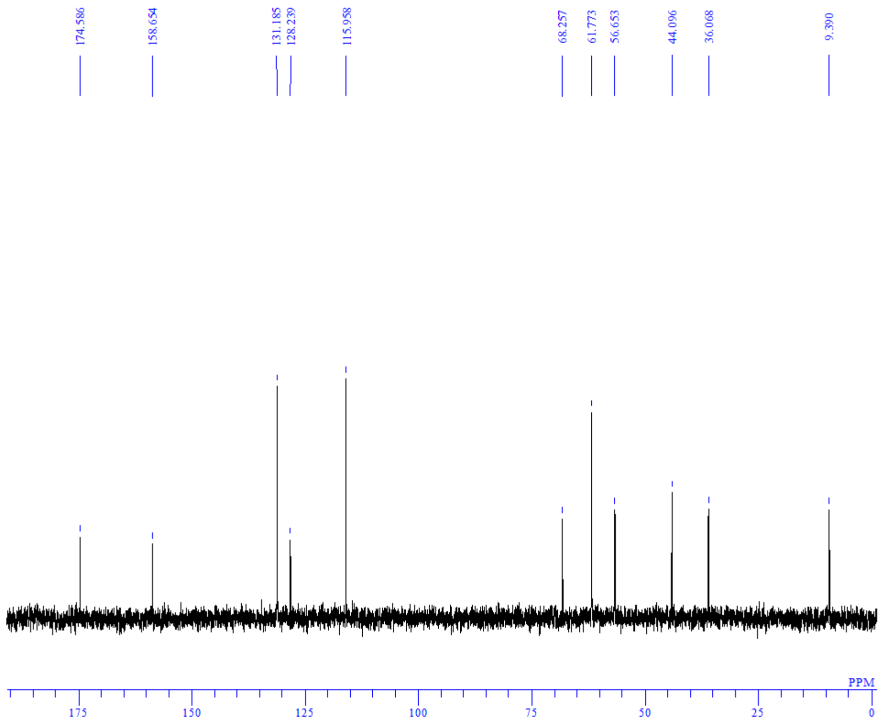

^{1}H-NMR and ^{13}C-NMR for compound 6

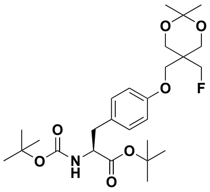

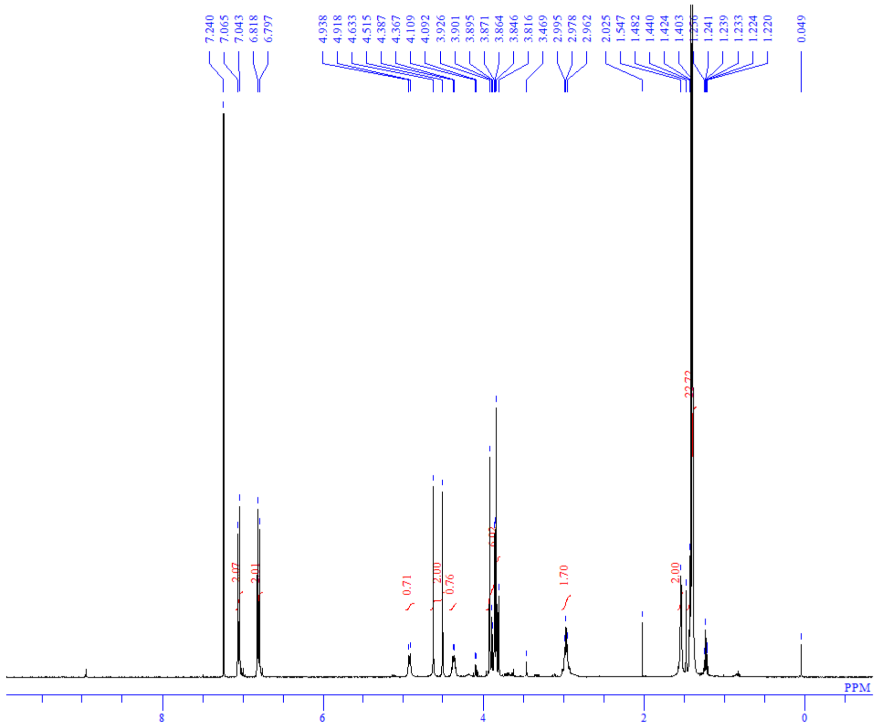

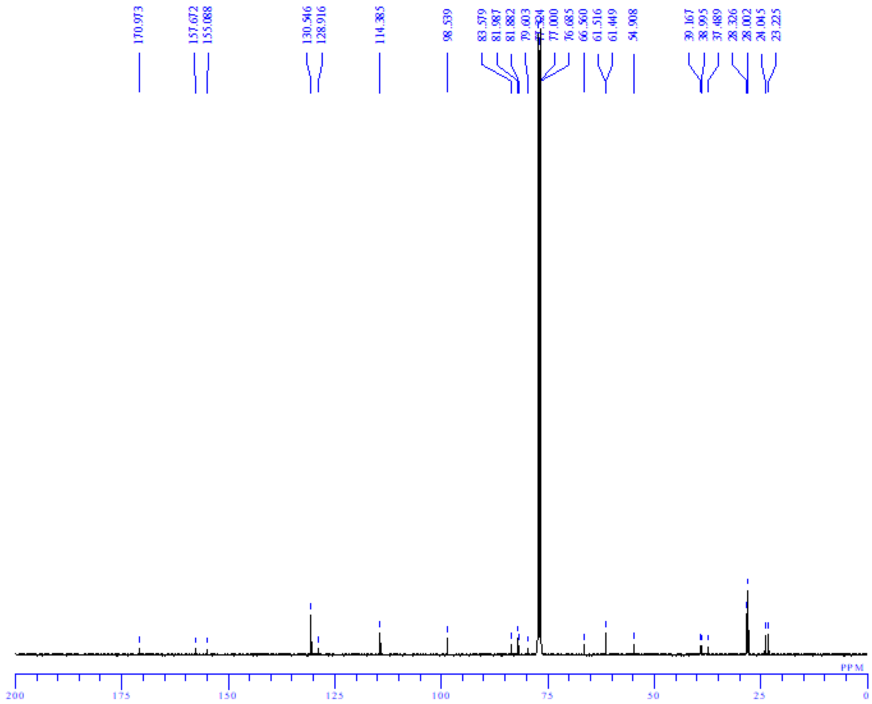

^{1}H-NMR, ^{13}C-NMR and ^{19}F-NMR for F-NpGT

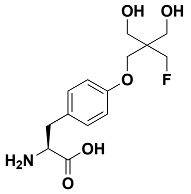

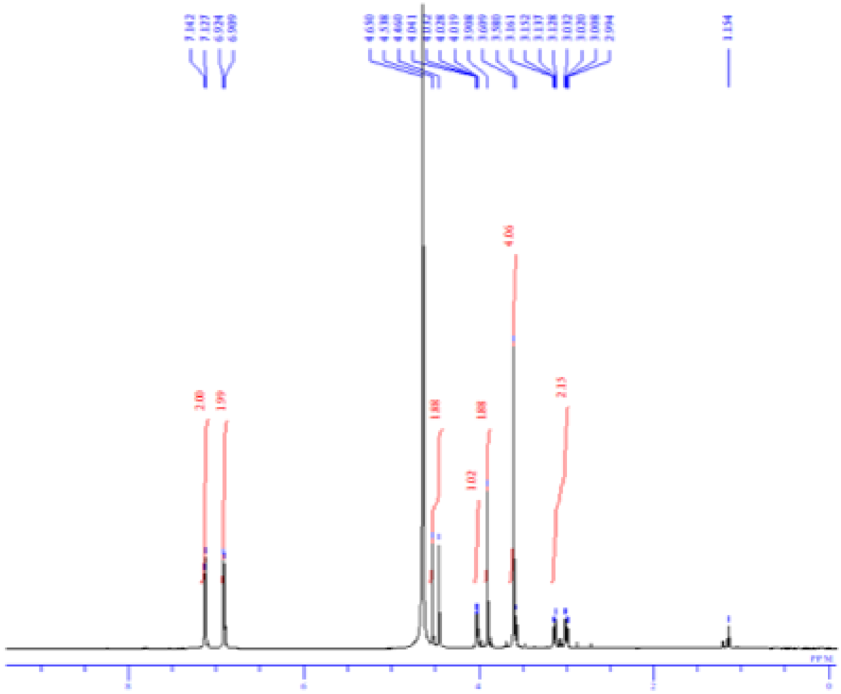

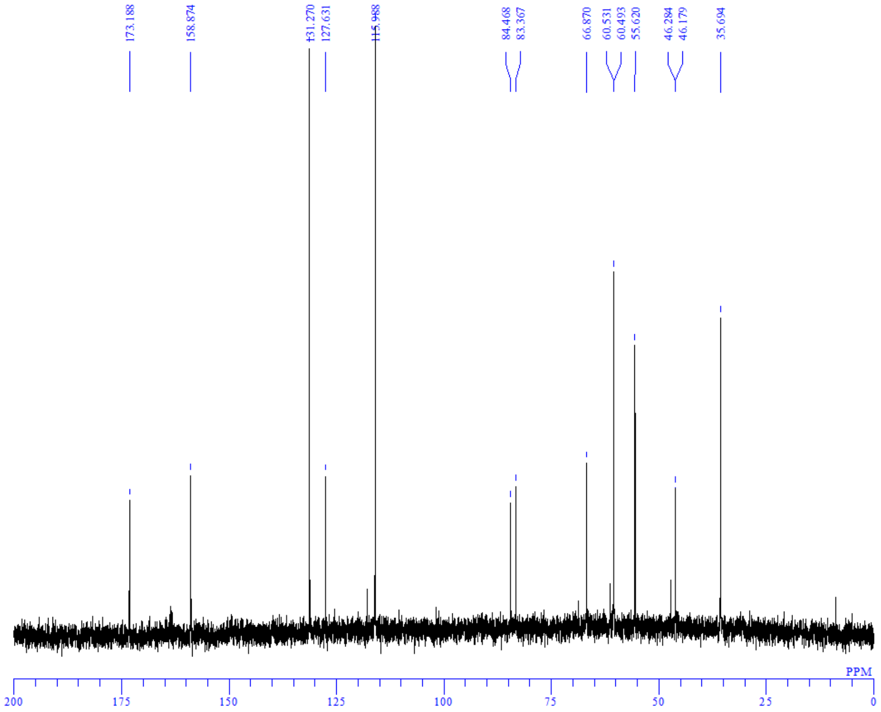

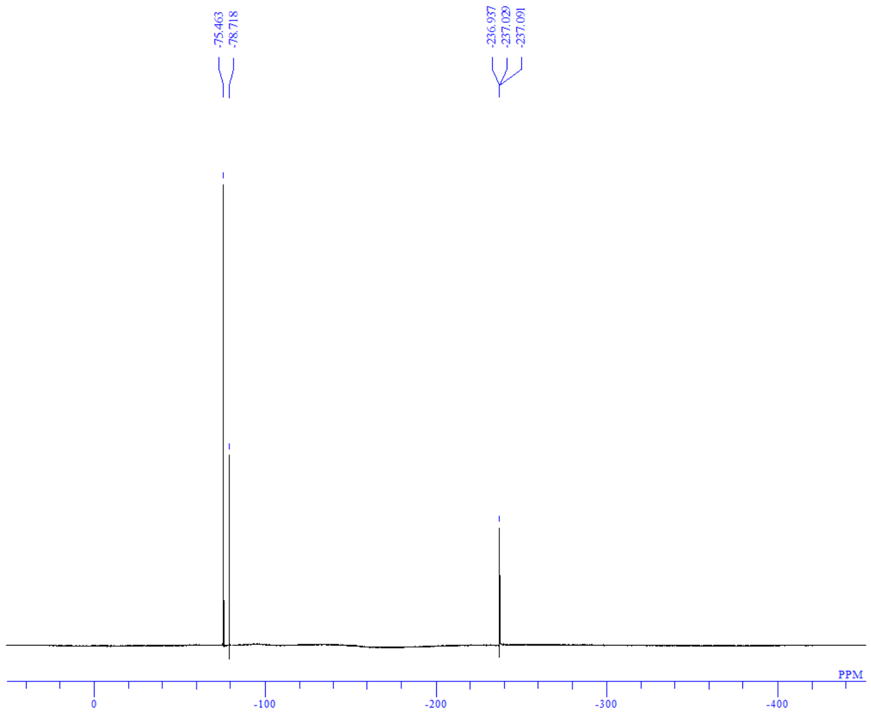
-75.463
-78.718
-236.937
-237.029
-237.091
PPM
0
-100
-200
-300
-400

Reference

1. Akizawa H, Imajima M, Hanaoka H, Uehara T, Satake S, Arano Y. Renal brush border enzyme-cleavable linkages for low renal radioactivity levels of radiolabeled antibody fragments. *Bioconjugate Chem.* 2013;24:291-299.
